# Influence of operating pressure on the durability of a satellite hydraulic motor supplied by rapeseed oil

**DOI:** 10.1038/s41598-024-61072-9

**Published:** 2024-05-07

**Authors:** Pawel Sliwinski

**Affiliations:** Faculty of Mechanical Engineering and Ship Technology (Division of Hydraulics and Pneumatics), Gdansk University of Technology, ul. Gabriela Narutowicza 11/12, 80-233 Gdańsk, Poland

**Keywords:** Hydraulic motor, Non-circular mechanism, Satellite mechanism, Energy efficiency, Oil, Life cycle, Discharge machining, Satellite, Rotor, Curvature, Engineering, Mechanical engineering

## Abstract

This article describes the results of a durability test of a hydraulic satellite motor supplied by rapeseed oil. The tests were carried out on a test stand in a power recuperation system. The tests of the motor were carried out at a constant shaft speed for three fixed pressure drops in the motor. This made it possible to demonstrate the influence of the motor operating pressure on the durability of the satellite mechanism. The influence of the pressure drop in the motor and the influence of the operating time on the motor absorbency, on the torque on the motor shaft and the influence on the volumetric and hydraulic-mechanical efficiency are also shown. The basic relationship between the efficiency of the motor and the temperature rise in the motor is also described. The results of the calculations of the temperature rise in the motor are compared with the experimental results. The article also shows which components of the motor’s working mechanism wear out the fastest. The cause of the wear and failure is also explained.

## Introduction

Hydraulic motors are executive organs in hydrostatic drive systems and their task is to convert hydraulic power into mechanical power^1–6^.Various liquids can be used to power hydraulic motors. Nowadays, when we talk about hydraulic drive systems, the default is to think of mineral oil as the working liquid. However, there are also hydraulic systems in which other liquids are used, such as oil-in-water emulsions (HFA), synthetic liquids (HFD) and water. Other environmentally friendly liquids are also used, such as vegetable oils or, more generally, hydraulic oils developed on the basis of vegetable oils. The parameters of these liquids, such as viscosity, density, lubricating properties and liquid cleanliness, etc., influence the energy conversion efficiency of hydraulic systems and their durability^7–17^.

In power hydraulics, vegetable oils are not yet as widely used as mineral oils. However, their properties have been systematically researched for some time in order to test the applicability of these oils as working liquids in hydrostatic systems^18–22^. Among the vegetable oils, rapeseed oil is the most widely used. This oil stands out from other vegetable oils because of its easy availability on the market and its affordable price (it is lower than that of mineral oil).

Hydraulic oils based on vegetable oils with a viscosity class of VG46 are known on the market. These oils are enriched with thermo-oxidising, depressurising, anti-foaming and lubricating additives^20^. They are therefore biodegradable vegetable oils with improved lubricating properties.

For over a dozen years now, developmental research has been carried out on a new generation of positive displacement hydraulic machines that can to work with different liquids, not only mineral oil, but also oil-in-water emulsion and pure water. These machines are satellite pumps and motors^22–36^. The characteristics of satellite pumps and motors operating with these liquids (and especially water) have already been described in detail and published, e.g. in^4,22,37–41^. Initial attempts were also made to test the properties of a satellite motor supplied with edible rapeseed oil (refined rapeseed oil). The first results are presented in^41,42^. However, these publications contain no more information on the effects of the load M (torque) on the absorption Q, the volumetric efficiency h_v_ and the pressure-mechanical efficiency h_hm_ as a function of the motor running time.

The literature on durability tests for other types of hydraulic motors is also sparse. In^43^, for example, it was written that the wear of the surfaces of the rotor of hydraulic planetary motors increases the gaps, resulting in a reduction in speed of up to 65% and torque of up to 42%. And in^44^, the change in the performance characteristics of the hydraulic planetary motor during operation was described for a certain range of changes in its operating parameters.

In order to obtain basic information on the durability of satellite mmotor supplied with liquids other than mineral oil, it was decided to carry out a further study on the durability of a hydraulic satellite motor supplied with edible rapeseed oil (refined rapeseed oil). It is generally known that this oil has poorer lubricating properties than mineral oil, but certainly better than water^20–22,45^. This will give you information on how the efficiency of a motor changes depending on its operating time at nominal parameters and how long this motor will last (to the point of damage or destruction). The results of the material tests and the failure mechanisms of the satellite mechanism teeth, i.e. pitting and cracking at the tooth root, have already been described in the article^46^.

The decision to test the hydraulic motor with edible vegetable oil was also made in order to assess its suitability and practicality for use in simple hydraulic systems operating indors, i.e. at ambient temperatures above zero.

## Tested motor

**Figure 1:**
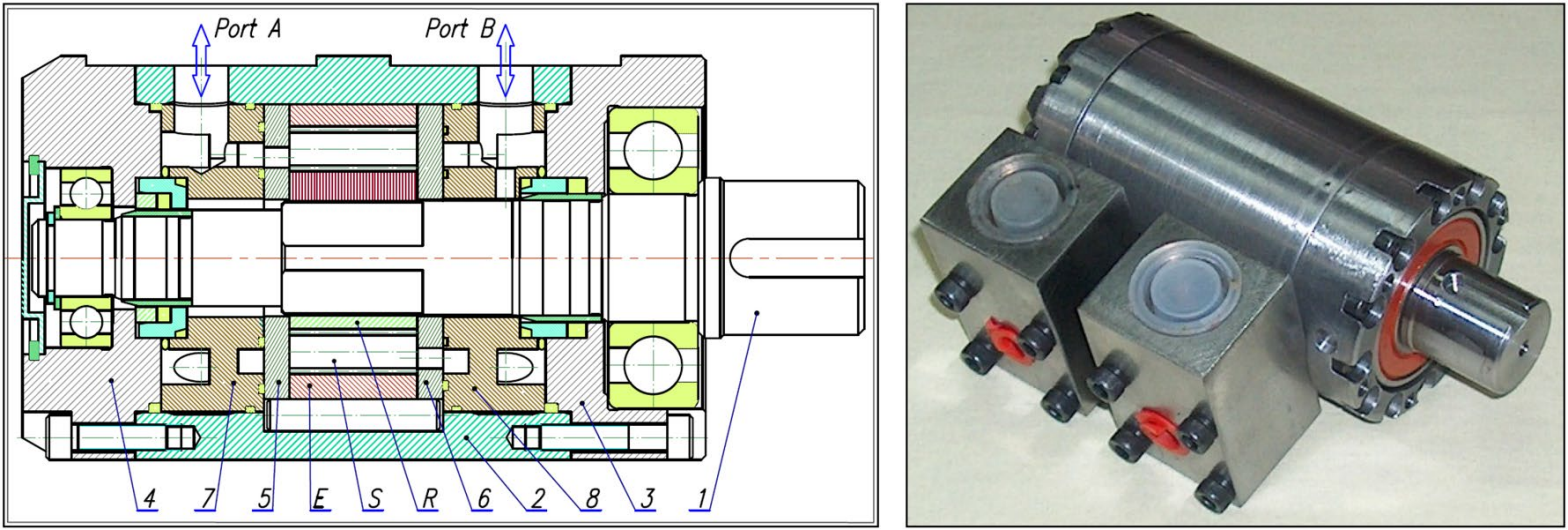
Tested motor (satellite motor)^22,37,38,46–48^: E—external gear (curvature), S—satellite, R—rotor, 1—shaft, 2,3 and 4—housing, 5 and 6—commutation plates (compensation plates), 7 and 8—inflow/outflow manifolds.

**Figure 2:**
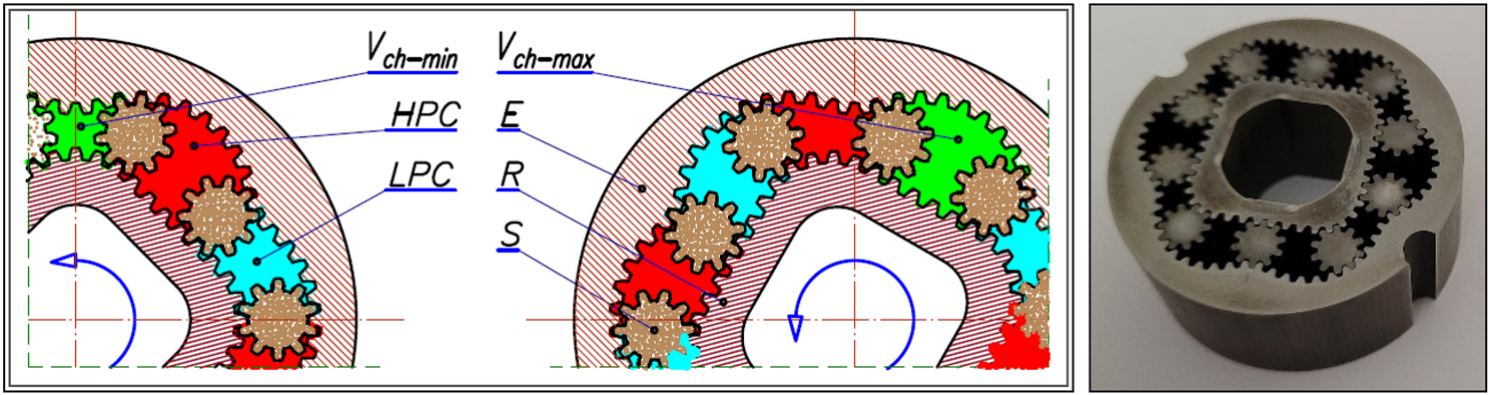
Satellite mechanism: E—external gear (curvature), R—rotor, S—satellite, HPC—high pressure chamber, LPC—low pressure chamber, V_ch-min_—working chamber with minimum volume, V_ch-max_—working chamber with maximum volume.

The research object was a prototype of a satellite hydraulic motor (Figs. 1 and 2). The operating principle of the satellite motor is generally known and is described in^22,47–49^.

**Figure 3:**
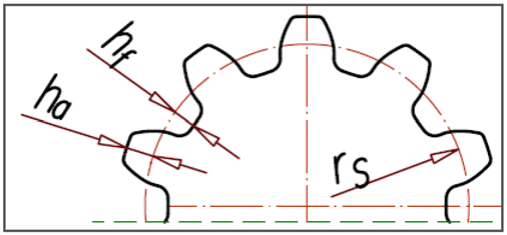
Basic parametres of the satellite: h_a_—addendum, h_f_—dedendum, r_S_—the reference radius.

The motor uses satellite mechanism type 4 × 6, i.e. the mechanism with a four-hump rotor (n_R_ = 4), a six-hump curvature (n_E_ = 6) and with ten satellites (Figs. 2 and 3). The theoretical working volume of the motor is q_t_ = 16,7 cm^3^/rev.

**Figure 4:**
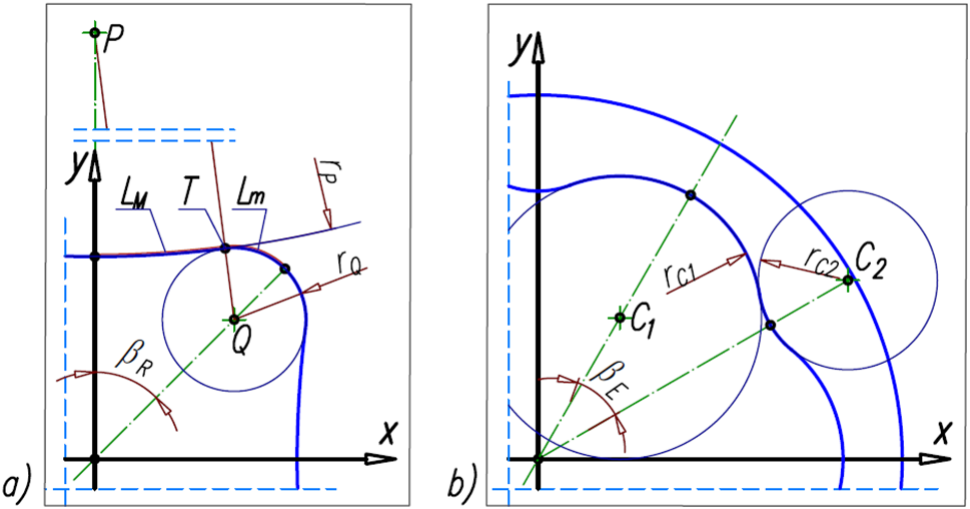
Geometry of the rotor of the tested satellite mechanism; r_C1_ and r_C2_—radii of the circles of curvature, L_M_—length of the arc with radius R_M_, L_m_—length of the arc with radius R_m_. Technical data in Table 1.

The publication^38^ states that the considered mechanism is a scaled copy of the mechanism with module 1.5 mm. The mechanism with module 1.5 mm was designed so that it can be produced using classical machining methods (chiselling and milling). The pitch line of the rotor consists of circles with radii r_P_ and r_Q_, which are connected at point T. The pitch line of the curvature was approximated by fragments of circles with radii r_C1_ and r_C2_^23^. A conceptual sketch of the rotor part of the satellite mechanism with the basic geometrical dimensions is shown in Fig. 4. The technical data of the satellite mechanism are listed in Table 1.

**Table 1:** Parameters of the tested satellite mechanism^38^. Symbols like in Figs. 3 and 4.

n_R_	n_E_	β_R_	β_E_	m	H
4	6	45°	30°	0.6 mm	20 mm

z_S_	z_R_	z_E_	r_S_	p_bs_	α_p_
10	44	66	3.0 mm	1.77 mm	20°

r_P_	x_P_	y_P_	r_Q_	x_Q_	y_Q_
202.2 mm	0.0 mm	213.78 mm	6 mm	5.657 mm	5.657 mm

x_T_	y_T_	L_M_	L_m_	h_a_	h_f_
6.895 mm	11.130 mm	5.508 mm	4.859 mm	0.26 mm	0.21 mm

r_C1_	x_C1_	y_C1_	r_C2_	x_C2_	y_C2_
9.333 mm	5.380 mm	9.319 mm	5.898 mm	20.333 mm	11.739

Other symbols: H—nominal height of the satellite mechanism, m—the tooth module, z_E_—the number of teeth on the external gear (curvature), z_R_—the number of teeth on the rotor, z_S_—the number of teeth on the satellite, α_p_—the pressure angle, p_bS_—the base pitch of the satellite teeth, x_P_, y_P_, x_Q_, y_Q_, x_T_, y_T_, x_C1_, y_C1_, x_C2_, y_C2_—coordinates of points P, Q, T, C_1_ and C_2_ (Fig. 4).

**Figure 5:**
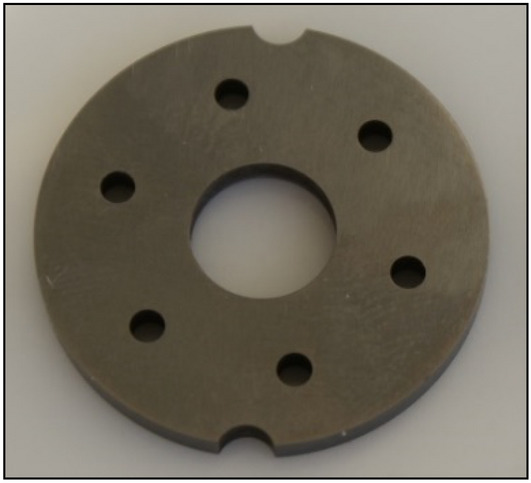
Commutation (compensation) plate of the satellite motor.

The non-core components of the satellite motor are the commutation plates (Fig. 1—items 5 and 6 and Fig. 5). They have the task of guiding the liquid into the working chambers of the motor and discharging it from them. Both plates are identical in design and manufacture.

The presented satellite mechanism was made of NIMAX steel (Table 2)^50^. This mechanism was manufactured using the WEDM (Wire Electrical Discharge Machining) process. After the WEDM process, the mechanism was nitrided.

**Table 2:** Basic parameters of the NIMAX steel^38,50^.

Density	Young module E	Poisson number v	Surface hardness after gas nitriding
7900 kg/m^3^	2.05⋅10^11^ Pa	0.3	950 MHV

Hardness	Yield strength Rp_0.2_	Tensile strength R_m_	Depth after gas nitriding
~ 370 HB	785 MPa	1265 MPa	0.25 mm

The article^46^ describes material tests for the satellite mechanism. It was shown that the average case hardness of nitrided steel NIMAX is 1100 HV0.1 (with a range of 1080HV to 1180HV). The depth of the hardened insert was 0.4 mm. The teeth of the satellite mechanism are therefore hardened over their entire thickness.

During one full rotation of the rotor (360° rotation), the number n_ch_ of cycles volume change of all working chambers is as follows^22,24,38^:

1 $${n}_{ch}={n}_{E}\cdot {n}_{R}$$

Publication^38^ concluded that for one full revolution of the rotor, i.e. for α_R_ = 360°:

- the number of contacts of each rotor tooth with the satellite tooth is i_RTS_ = 6;
- the number of contacts of each curvature tooth with the satellite tooth is i_ETS_ = 4.

Based on the theoretical analyses, presented in the publication^38^, it can also be concluded that the total number i_S_ of contacts satellite teeth with the rotor teeth and the curvature for one full revolution of the rotor (for α_R_ = 360°) is:

2 $${i}_{S}=0.2\cdot {z}_{E}\cdot \frac{{n}_{R}}{{n}_{E}+{n}_{R}}$$

Thus, for the 4 × 6 type satellite mechanism i_S_ = 5.28. In addition, it was shown in^38^ that for nitrided NIMAX steel, the contact fatigue strength of gears σ_Hlim_ ≈ 1250 MPa, but the value of allowable normal stress in gears is maximally (in a very optimistic variant) σ_per_ ≈ 2500 MPa. In^38^ it was also shown that for Δp_i_ = 25 MPa the stresses in the teeth:

- are not exceeded if the mechanism is backlash-free and therefore favourable values for the K and Z factors are asssumed in the stress calculation formulae;
- are exceeded by up to 60% (assuming unfavourable values for the K and Z factors in the calculations).

## Working liquid

**Figure 6:**
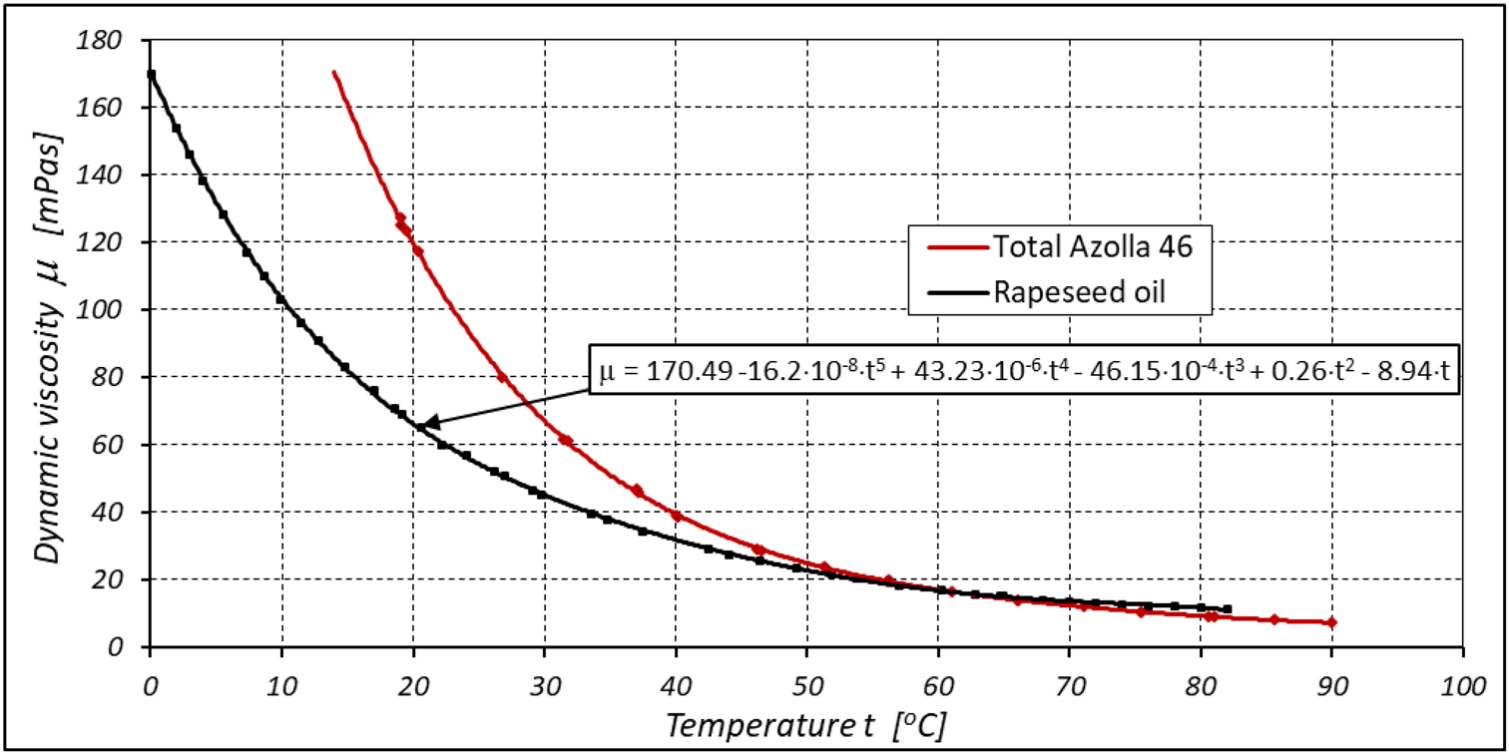
Dynamic viscosity µ of refined rapeseed oil (edible oil) and Total Azolla 46 mineral oil.

**Figure 7:**
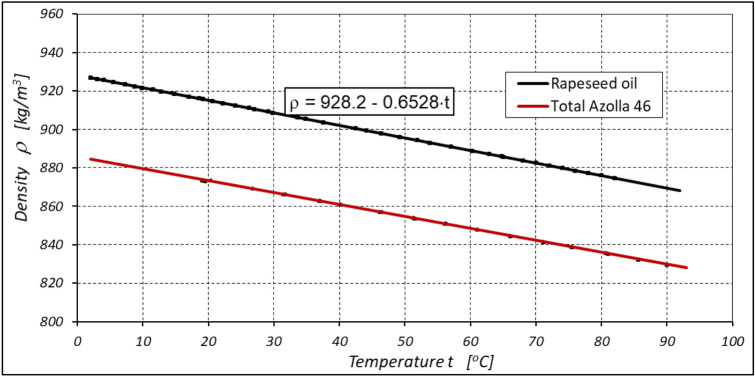
Density ρ of refined rapeseed oil (edible oil) and Total Azolla 46 mineral oil.

Refined rapeseed oil (edible oil) was used as the working liquid in the system. The characteristics of dynamic viscosity µ and density ρ of this oil are shown in Figs. 6 and 7. For comparison, the viscosity µ and density ρ characteristics of the mineral oil Total Azola ZS 46 are also shown in these figures. It is clear that the density ρ of the oil decreases with increasing temperature t. The density ρ of rapeseed oil can be described by the following empirical formula (Fig. 7):

3 $$\rho =928.2-0.653\cdot t \left[\frac{kg}{{m}^{3}}\right]$$

However, the specific heat c of rapeseed oil is somewhat lower than the specific heat of mineral oil. For rapeseed oil $c=1850\frac{J}{kg\cdot K}$, but for mineral oil $c=1880\frac{J}{kg\cdot K}$ (for comparison, the specific heat of water $c=4180\frac{J}{kg\cdot K}$^51^.

The results of the lubricating properties test described in^22,45^ show that the limit seizure pressure p_oz_ for refined rapeseed oil is p_oz_ = 287.5 MPa and for mineral oil Total Azolla ZS 46 is p_oz_ = 386.4 MPa. Rapeseed oil therefore has poorer lubricating properties than mineral oil. The difference in the seizure pressure limit values is up to 25%^22,45^.

## Test rig

**Figure 8:**
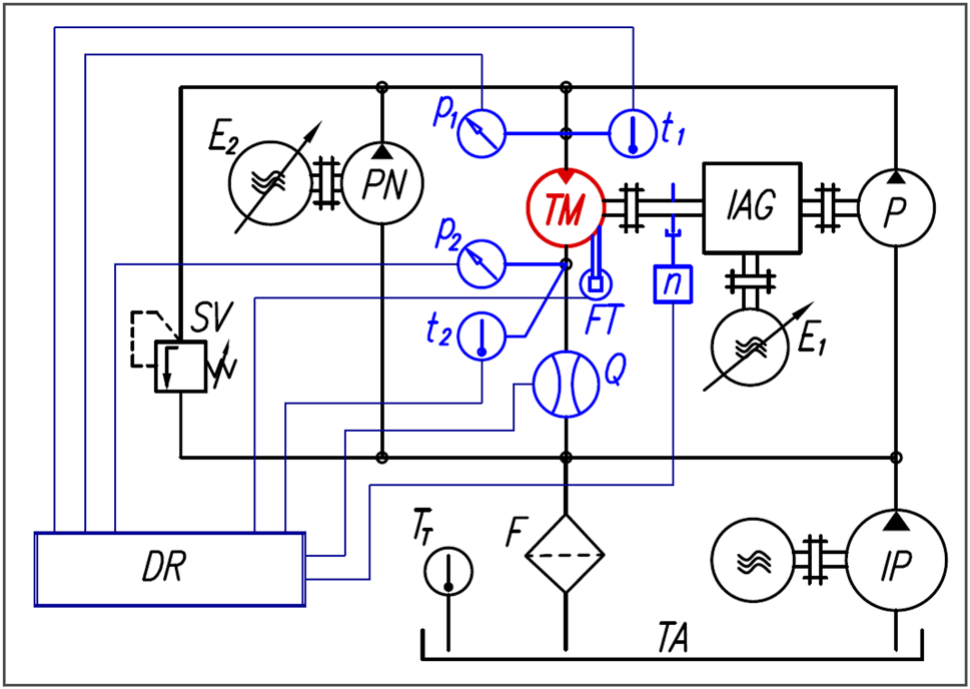
The hydraulic and measuring system of the test rig: P—pump, TM—tested motor, PN—pump for filling leaks in P and M, IP—impeller pump, SV—safety valve, F—filter, TA—tank, IAG—intersecting axis gear, E_1_ and E_2_—electric motors with frequency converters, DR—data recorder, p_1_, p_2_—pressure transducers, t_1_, t_2_, t_T_—temperature transducers, Q—flowmeter, FT—force transducer for torque M measurement, n—inductive sensor for rotational speed measurement,

**Figure 9:**
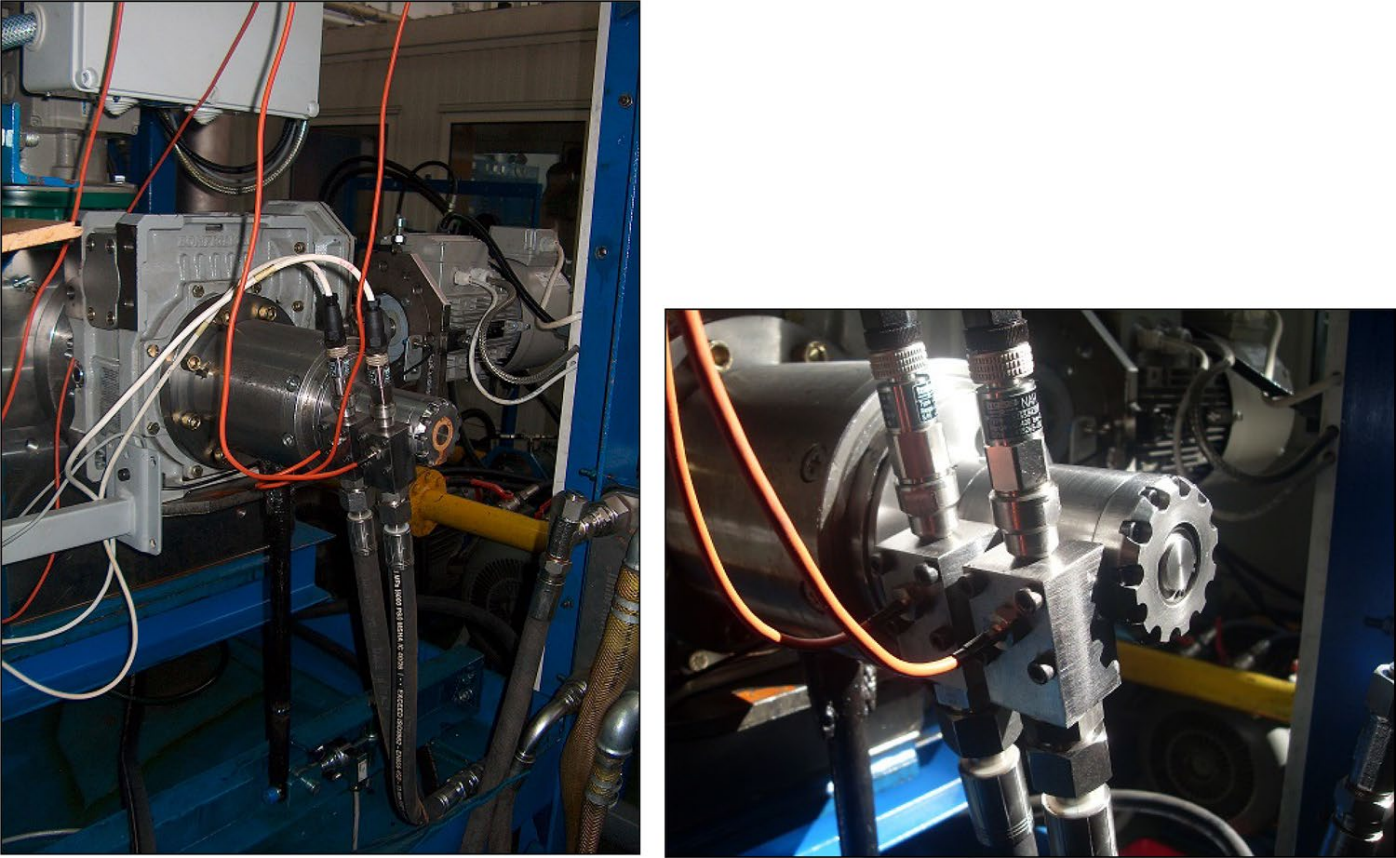
View of part of the test rig (left) with the satellite motor mounted for the test (right).

The durability test of the satellite motor was carried out on the test rig shown in Figs. 8 and 9. This test rig is equipped with an energy recovery and has been presented in many publications, including^37,39,40,47–49^. It should be made clear at this point that the hydraulic system of the thest rig was cleaned and flushed before the tests. The tank was filled with 500 L of rapeseed oil.

The following parameters were measured during the motor test:

- pressure p_1_ in the inlet port (strain gauge pressure transducer, range 0 ÷ 25 MPa, class 0.3);
- pressure p_2_ in the exaust port (strain gauge pressure transducer, range 0 ÷ 2.5 MPa, class 0.3);
- motor absorbency Q (mass flowmeter, range 0 ÷ 33 l/min, class 0.1);
- torque M (strain gauge force transducer FT mounted on the arm 0.5 m (the arm is fixed to the motor body), range 0 ÷ 100 N, class 0.1);
- rotational speed n of the motor shaft (inductive sensor, accuracy of measurement ± 0.01 rpm);
- temperatures t_1_ and t_2_ of liquid in the inlet and exaust ports of the motor (RTD temperature sensor, class A, max. error 0.5 °C).

On the test rig mentioned above, it is very easy to keep the constant pressure drop Δp measured in the motor ports (by adjusting the pump speed PN and thus its capacity accordingly—Fig. 8). This pressure drop is:

4 $$\Delta p={p}_{1}-{p}_{2}$$

If Δp = const. then torque M, measured at the motor shaft, is^40^:

5 $$M=\frac{{q}_{t}\cdot {\Delta p}_{i}}{2\cdot \pi }-{M}_{L}$$

where: q_t_—the theoretical working volume of the motor, M_L_—the torque of the mechanical losses, Δp_i_—the pressure drop in the working chambers of the motor^49^: ${\Delta p}_{i}=\Delta p-{\Delta p}_{ich}$(6), Δp_ich_—the pressure drop in the internal channels of the motor.

The method for determining Δp_ich_ is described in detail in^49^. Thus, the pressure drop Δp_i_ that load the working mechanism is smaller than the pressure drop Δp measured in the motor ports. In this way Δp_i_ influences the wear of the working mechanism.

## Relationship between the efficiency of the motor and the temperature rise in the motor

Every positive displacement machine (pump or motor) is characterised by energy losses. The amount of power N_L_ lost in the motor is calculated as follows:

7 $${N}_{L}=Q\cdot \Delta p-\frac{\pi }{30}\cdot M\cdot n$$

where: Q—the motor absorbency, n—rotational speed of the motor shaft.

The power N_L_ lost in the hydraulic motor influences the temperature rise of the motor components and the temperature rise Δt of the liquid in the motor ports. The heat flow from the liquid to the surroundings through the motor components is very low compared to the heat flow in the liquid. Therefore, the heat flow from the liquid to the surroundings is ignored in the considerations and it is assumed that the entire power lost N_L_ is absorbed by the liquid^52^. It can therefore be written that:

8 $${N}_{L}=\rho \cdot Q\cdot c\cdot {\Delta t}_{\mu }$$

where: Δt_µ_—the temperature rise of the liquid due to energy losses (depending on the viscosity µ of the liquid), c—the specific heat of the oil, ρ —the density of the oil.

In addition to the temperature rise Δt_µ_ of the liquid, there is a temperature drop Δt_ai_ which is associated with the pressure drop Δp of the liquid in the motor and is calculated as follows^52^:

9 $${\Delta t}_{ai}=k\cdot \Delta p$$

where k is the constant that depend on the initial temperature of the liquid. For mineral oil at t_1_ = 40 ÷ 50 °C k = 0.128 °C /MPa can be assumed^52^. The value of the constant k for rapeseed oil is not known and will be the subject of further analyses.

The temperature rise Δt in the motor ports is therefore:

10 $$\Delta t={{\Delta t}_{\mu }-\Delta t}_{ai}$$

A comparison of formulae (7) and (8) shows that:

11 $${\Delta t}_{\mu }=\frac{1}{\rho \cdot c}\cdot \left(\Delta p-\frac{\pi }{30}\cdot M\cdot \frac{n}{Q}\right)$$

Considering that the total efficiency η_c_ of a motor is defined as:

12 $${\eta }_{c}=\frac{\pi }{30}\cdot \frac{M}{\Delta p}\cdot \frac{n}{Q}={\eta }_{v}\cdot {\eta }_{hm}$$

then:

13 $${\Delta t}_{\mu }=\frac{\Delta p}{\rho \cdot c}\cdot \left(1-{\eta }_{v}\cdot {\eta }_{hm}\right)$$

where: η_v_—the volumetric efficiency of the motor; η_hm_—the pressure-mechanical efficiency of the motor.

Substituting formula (9) and (13) into formula (10) finally results in the following formula:

14 $$\Delta t=\Delta p\cdot \left(\frac{1}{\rho \cdot c}\cdot \left(1-{\eta }_{v}\cdot {\eta }_{hm}\right)-k\right)$$

It can be seen from this that if the efficiency η_c_ of the motor decreases, the temperature rise Δt in the motor is greater. If the value of the constant k is not known, it can be determined by transforming Eq. (13), namely:

15 $$k=\frac{1}{\rho \cdot c}\cdot \left(1-{\eta }_{v}\cdot {\eta }_{hm}\right)-\frac{\Delta t}{\Delta p}$$

The values of Δt, Δp, η_v_ and η_hm_ in the above formula were determined from experimental data. However, it is known that the efficiencies η_v_ and η_hm_ depend on the pressure drop Δp in the motor^39,40,49^. In order to obtain a more reliable result, it is therefore proposed to calculate the constant k as:

16 $$k=\frac{1}{n}\cdot \sum_{i=1}^{n}{k}_{\left(i\right)}$$

where: k_(i)_—the value of constant k calculated according to formula (15) for the i-th pressure drop Δp in the motor; n—the number of values of Δp and thus the number of calculated values of the constant k_(i)_.

## Conditions for conducting the experiment

Three series of durability tests of the satellite motor were performed. A new working mechanism was maintained was used in each series of tests. In each series of tests, a constant pressure drop Δp was maintained in the motor, i.e.:

1. the first series—at Δp = 20 ± 0.5 MPa;
2. the second series—at Δp = 15 ± 0.5 MPa;
3. the third series—at Δp = 10 ± 0.5 MPa.

The motor tests were carried out at the one constant speed n = 1500 ± 1 rpm (in all three series).

The liquid temperature was not stabilised in the test rig, as can be seen in detail from the temperature characteristics in the following sections. When, the system was started, the temperature was therefore significantly lower than during the test. In this way, the actual operating conditions of the motor in the industrial equipment were reflected.

**Figure 10:**
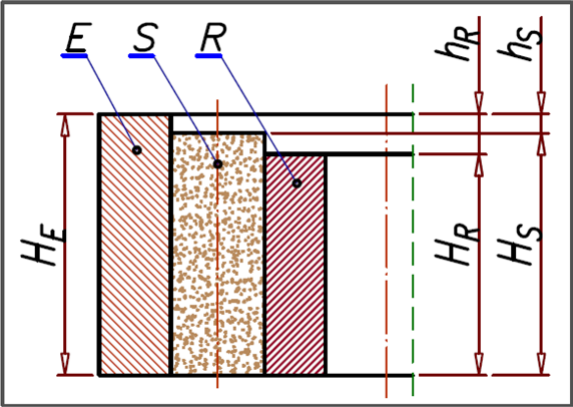
The height H_E_ of the curvature, the axial clearance h_S_ of the satellite, the axial clearance h_R_ of the rotor.

**Figure 11:**
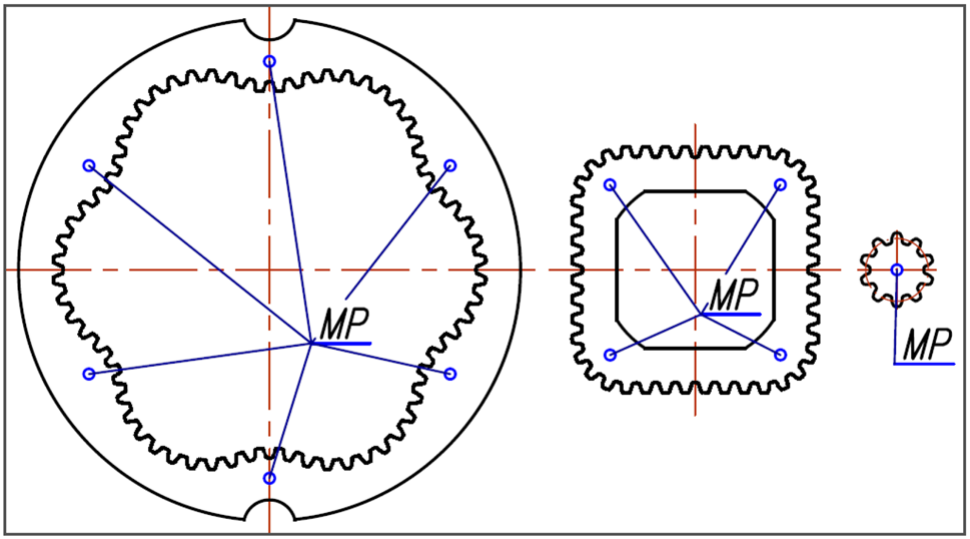
Points MP for measuring the height H_E_ of the curvature, the height H_R_ of the rotor and the height H_S_ of the satellite.

Before the new satellite mechanism was installed to the motor, the heights of the mechanism’s components were measured at the MP points as shown in Figs. 10 and 11. The mass of these elements was also measured. The height H_E_ of the curvature is the average value of the measurements at the six points. Similarly, the height H_R_ of the rotor was calculated (as the average of four measurements).

The axial clearance h_R_ of the rotor was calculated as:

17 $${h}_{R}={H}_{E}-{H}_{R}$$

and the axial clearance h_S_ of the satellites was calculated as:

18 $${h}_{S}={H}_{E}-\frac{\sum_{i=1}^{i={n}_{R}+{n}_{E}}{H}_{Si}}{{n}_{S}}$$

where: H_E_—the height of the curvature, H_R_—the height of the rotor. H_Si_—the height of the i-th satellite. n_S_—the number of satellites.

The values for the height of the components of the mechanism, their masses and the values of the axial clearance of the rotor and the satellites are presented in the next section.

In each series, the tests were carriedout in an intermittent cycle, i.e. the test stand was switched off from time to time. The test stand was also switched off at the end of the working day. The interruption of the test is represented by a vertical orange dashed line in each test drawing (in the following section). In addition, the motor was disassembled from time to time, the mechanism was cleaned, wear products were removed and the heights of the mechanism components and their weights were measured. The removal of wear products from the surfaces of the mechanism components required lapping of these surfaces. At the same time, they were lapped so as not to reduce the original height of these components. The values h_R_ and h_S_ were calculated according to the formulae (17) and (18). In each figure showing the results of the study (figures in the following section), the time at which the disassembly was performed is illustrated with a vertical blue dashed line.

In order to objectively compare the wear, the relative weight loss of the components was calculated using the following formula:

19 $${\delta m}_{S}=100\cdot \left(1-\frac{\sum_{i=1}^{i={n}_{S}}{m}_{Si}}{\sum_{i=1}^{i={n}_{S}}{m}_{Si(t=0)}}\right)$$

20 $${\delta m}_{E}=100\cdot \left(1-\frac{{m}_{E}}{{m}_{E(t=0)}}\right)$$

21 $${\delta m}_{R}=100\cdot \left(1-\frac{{m}_{R}}{{m}_{R(t=0)}}\right)$$

where: m_Si(t=0)_, m_R(t=0)_, m_E(t=0)_ —the initial mass of the satellite, the rotor and the curvature (for t = 0 min.); m_Si_, m_R_, m_E_—the mass of the salitellite, the rotor and the curvature during the test.

The experimental data corresponding to the set-up time τ_s_, i.e. the time to start and stop the hydraulic and measurement system, the time to set the parameters, etc., are not shown in the test results (tables and figures). During this time the motor was operated at a speed in the range of 0 ÷ 1500 rpm and in the pressure drop range Δp corresponding to the each series of measurements, i.e. Δp = 20, 15 and 10 MPa respectively. It was estimated that the time of unstabilised motor operation was:

1. in the first series (at Δp = 20 MPa)—about 40 min;
2. in the second series (at Δp = 15 MPa)—about 30 min;
3. w trzeciej serii (dla at Δp = 10 MPa)—about 52 min.

and his mean rotational speed is about n_m_ = 750 rpm.

## Results of the tests

### *Results of the test at Δp* = *20 MPa*

Table 3 shows the mass of the components of the satellite mechanism, the height H_E_ of the curvature and the average values h_R_ of the axial clearances of the rotor and the average values h_S_ of the axial clearances of the satellites measured before, during and after the tests.

**Table 3:** Mass, height and clearances of the componensts of the satellite mechanism—test for Δp = 20 MPa. Symbols H_E_, h_R_ and h_S_ like in Fig. 10.

		m_E_ (g)	m_R_ (g)	m_S_ [g]	H_E_ (mm)	h_R_ (µm)	h_S_ (µm)
Time τ (min)	0	99.813	43.104	4.536	19.998	4.5	6.2
	11.5	99.801	43.103	4.535	20.007	6.5	6.45
	229	99.788	43.075	4.533	20.009	7.0	7.1
	765	99.781	43.010	4.529	20.011	7.0	9.1
	1677	99.741	42.745	4.521	20.010	7.5	8.35
	2135	99.726	damage	4.516*	20.019	damage	7.75*

m_S_—the average mass of the satellites, m_E_—the mass of the curvature, m_R_—the mass of the rotor.

*Measurement for only four satellites (6 were broken).

**Figure 12:**
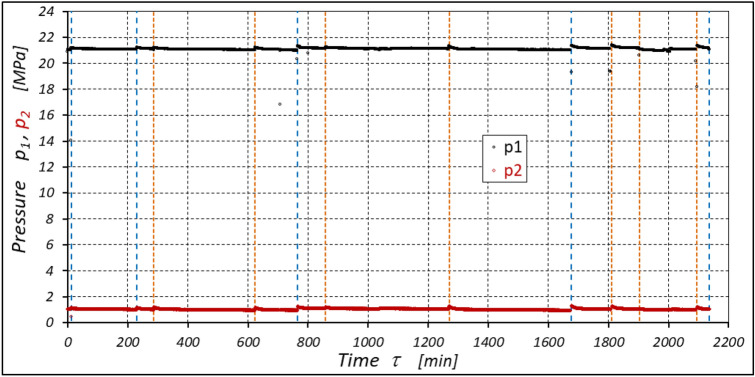
Characteristics of the pressure p_1_ and p_2_ in the motor ports as a function of time τ for Δp = 20 MPa and n = 1500 rpm.

**Figure 13:**
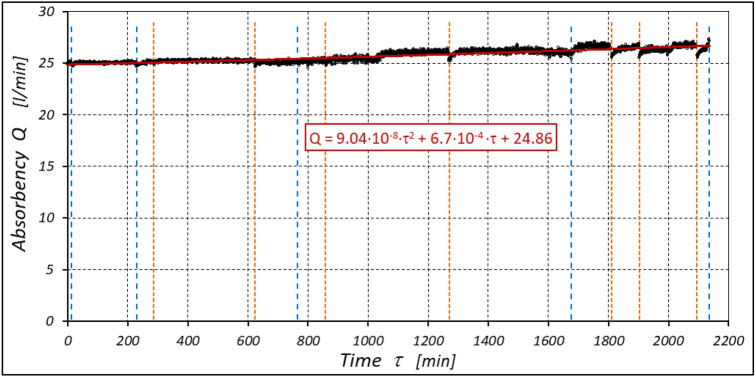
Characteristics of the motor absorbency Q as a function of time τ for Δp = 20 MPa and n = 1500 rpm.

**Figure 14:**
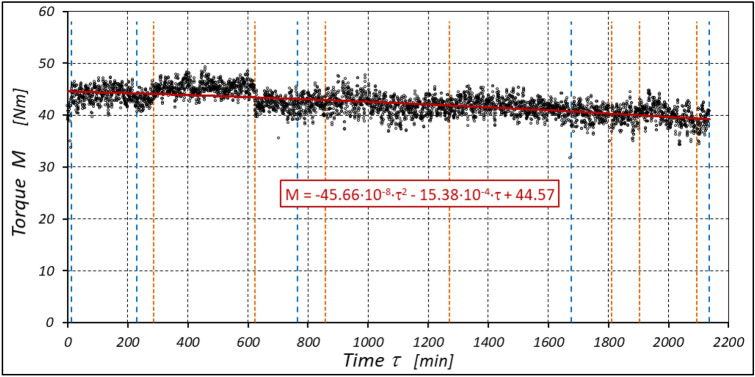
Characteristics of the torque M as a function of time τ for Δp = 20 MPa and n = 1500 rpm.

**Figure 15:**
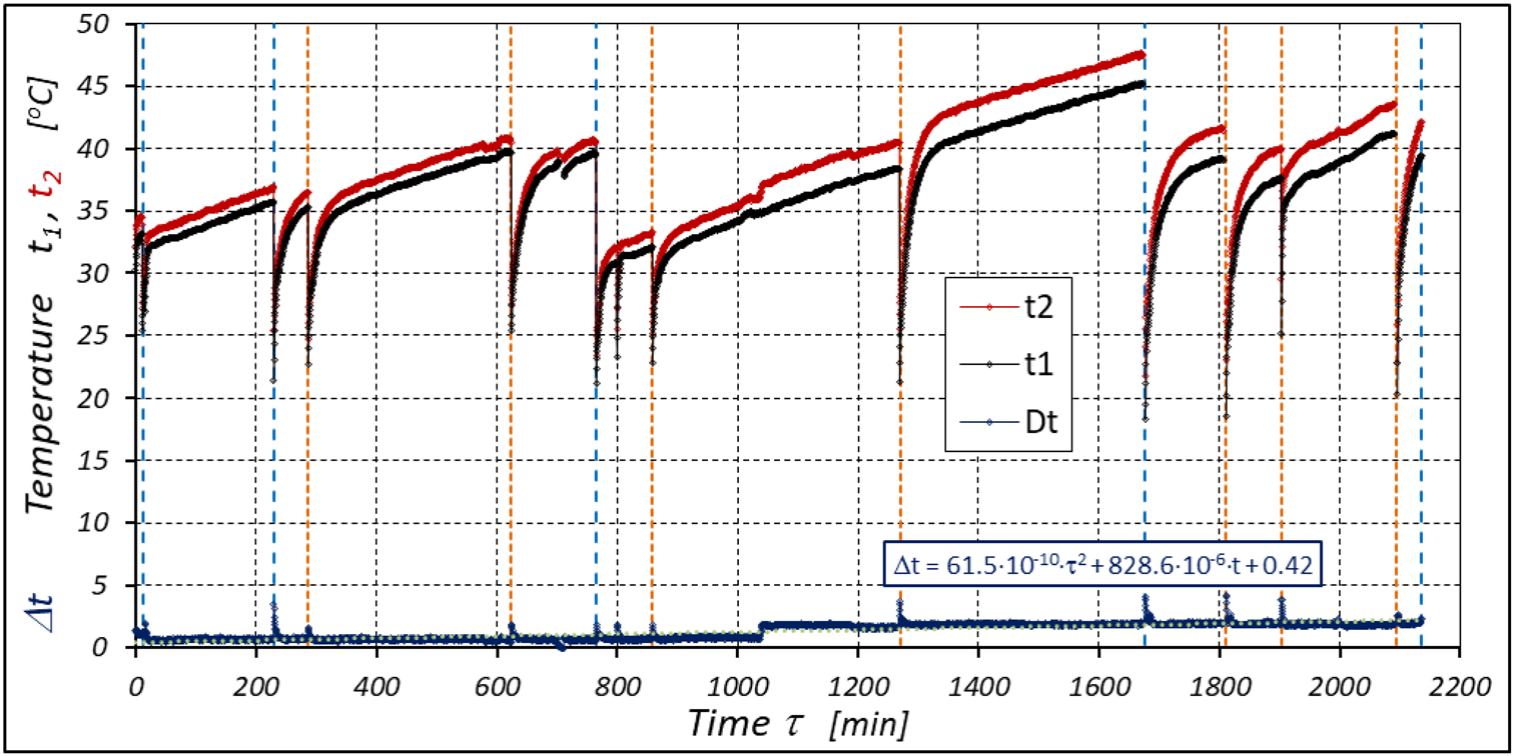
Characteristics of the temperatures t_1_ and t_2_ in the motor ports as a function of time τ for Δp = 20 MPa and n = 1500 rpm.

The results of measurements of the pressures p_1_ and p_2_, the absorption Q, the torque M and the temperatures t_1_ and t_2_ in the motor ports are shown in Figs. 12, 13, 14 and 15.

**Figure 16:**
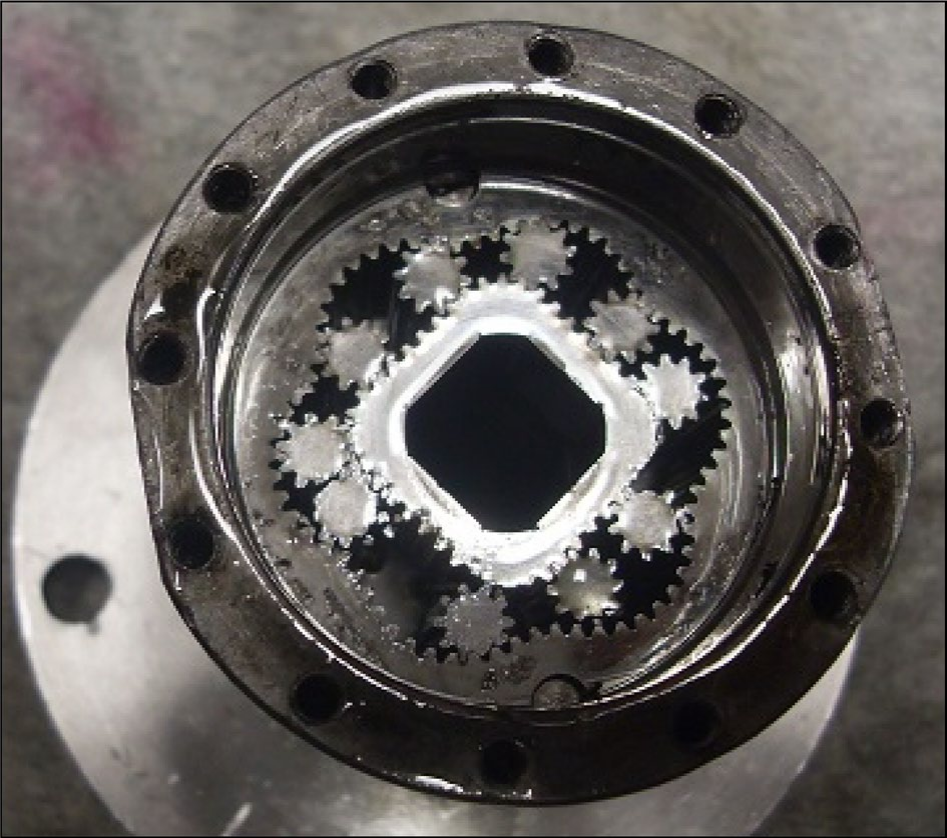
The destroyed satellite mechanism.

After 2135 min of operation, the satellite mechanism was destroyed (Fig. 16).

**Figure 17:**
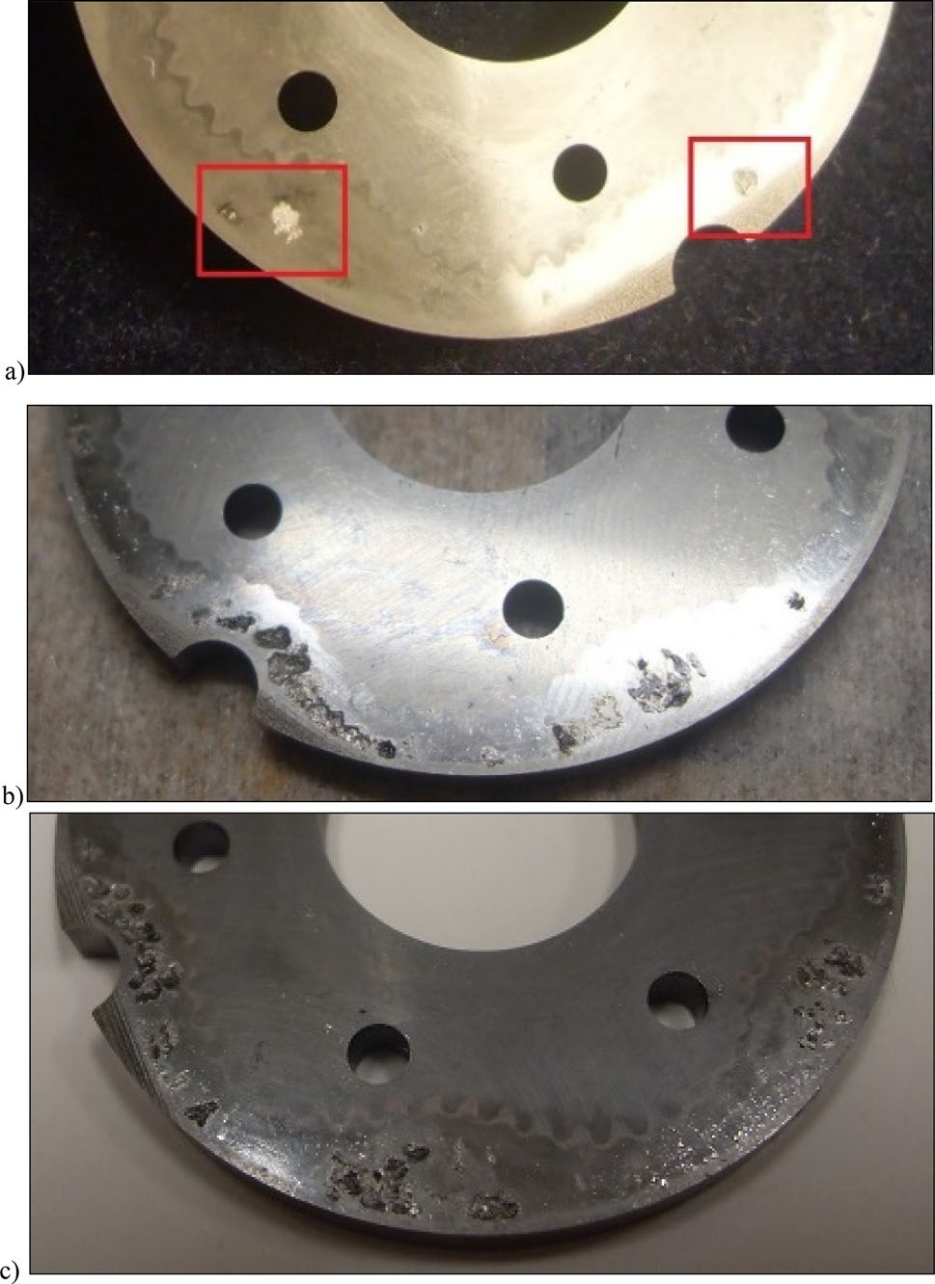
Fretting on the commutation plate: (**a**) after 11.5 min of operation; (**b**) after 1667 min of operation, (**c**) after completion of the test.

**Figure 18:**
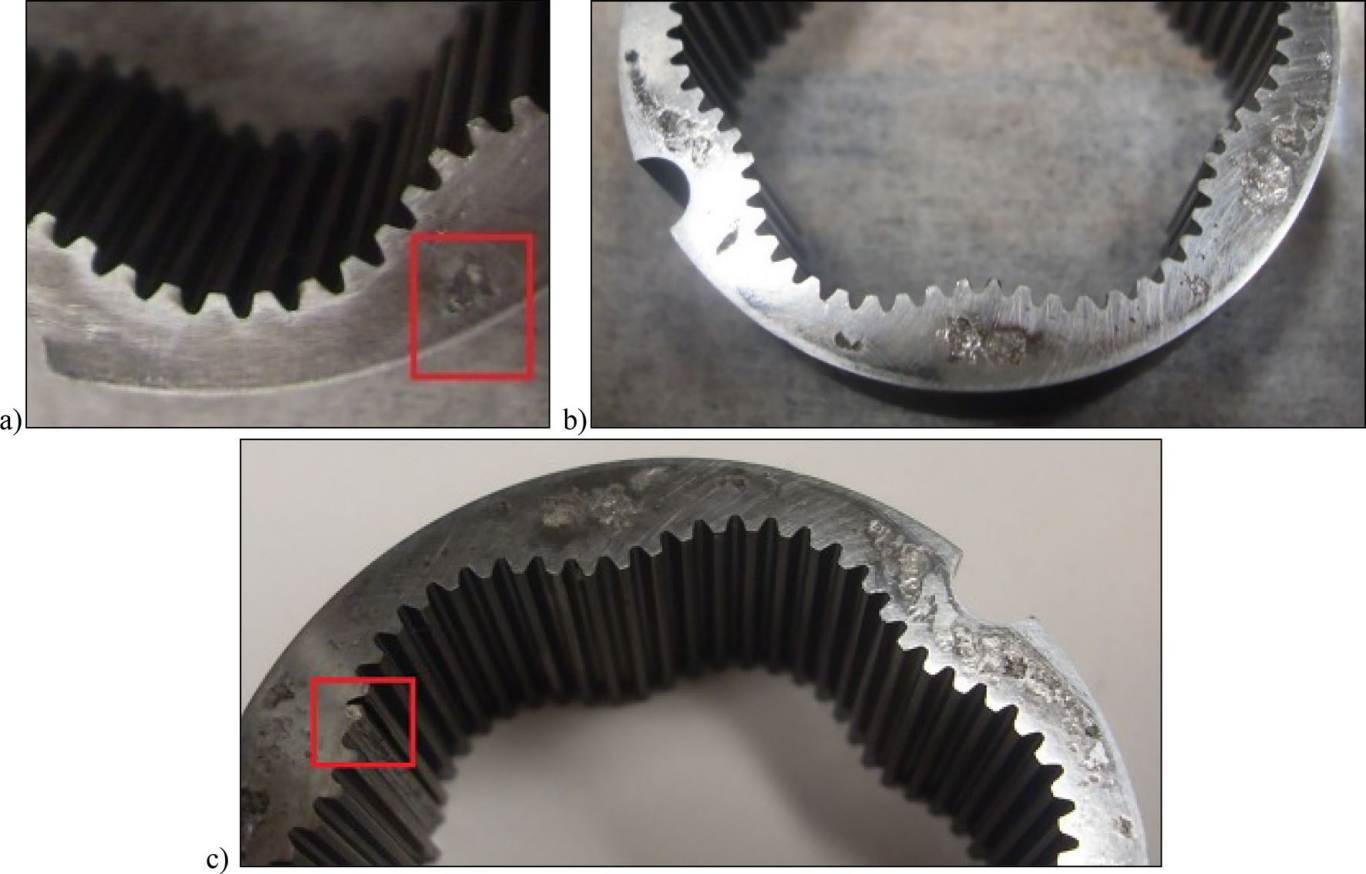
Fretting on the curvature: (**a**) after 11.5 min of operation; (**b**) after 1667 min of operation, (**c**) after completion of the test.

**Figure 19:**
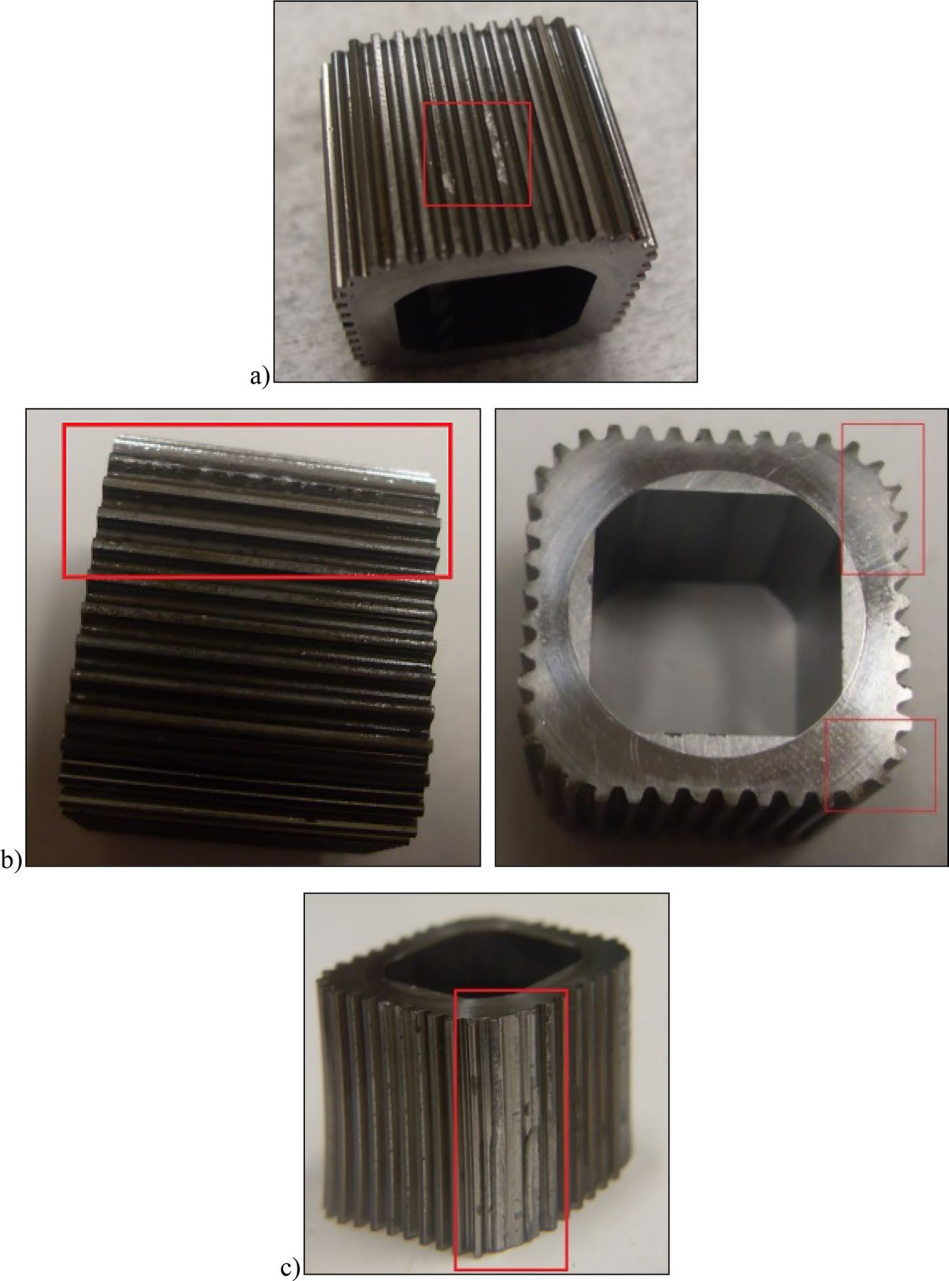
Photos of the rotor: (**a**) after 765 min of operation; (**b**) after 1667 min of operation, (**c**) after completion of the test.

Photographs of the satellite mechanism components during and after the test are shown in Figs. 17, 18 and 19.

### *Results of the test at Δp* = *15 MPa*

Table 4 shows the mass of the components of the satellite mechanism, the height H_E_ of the curvature and the average values h_R_ of the axial clearances of the rotor and the average values h_S_ of the axial clearances of the satellite measured before, during and after the tests.

**Table 4:** Mass, height and clearances of the componensts of the satellite mechanism—test for Δp = 15 MPa. Symbols H_E_, h_R_ and h_S_ like in Fig. 10.

		m_E_ (g)	m_R_ (g)	m_S_ (g)	H_E_ (mm)	h_R_ (µm)	h_S_ (µm)
Time τ (min)	0	99.849	43.168	4.536	20.002	5.15	4.50
	550	99.832	43.139	4.532	20.008	7.21	8.15
	1415	99.804	43.095	4.526	20.010	10.18	8.86
	2690	99.738	43.007	4.518	20.013	11.02	9.03
	3092.5	99.829	42.985	4.526	20.014	12.00	9.00

**Figure 20:**
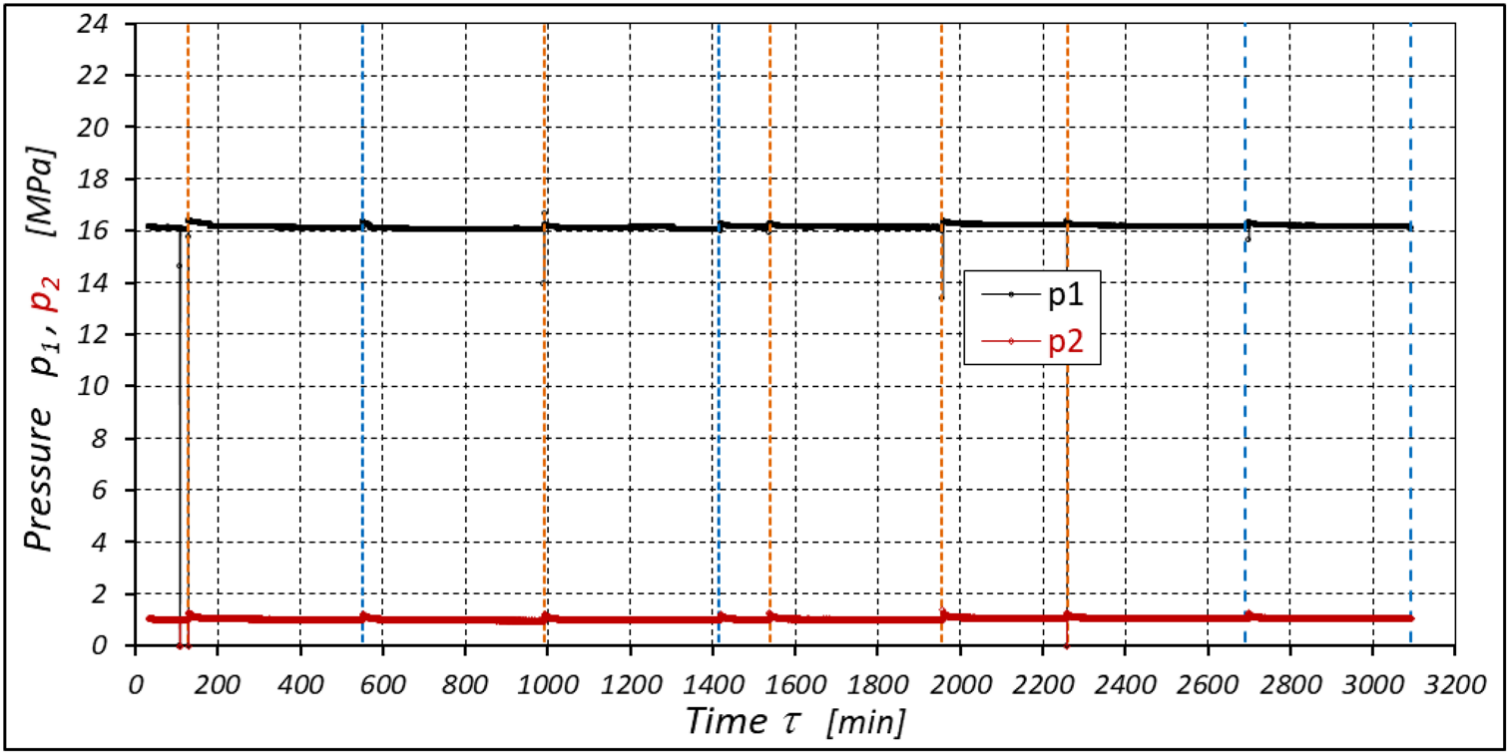
Characteristics of the pressure p_1_ and p_2_ in the motor ports as a function of time τ for Δp = 15 MPa and n = 1500 rpm.

**Figure 21:**
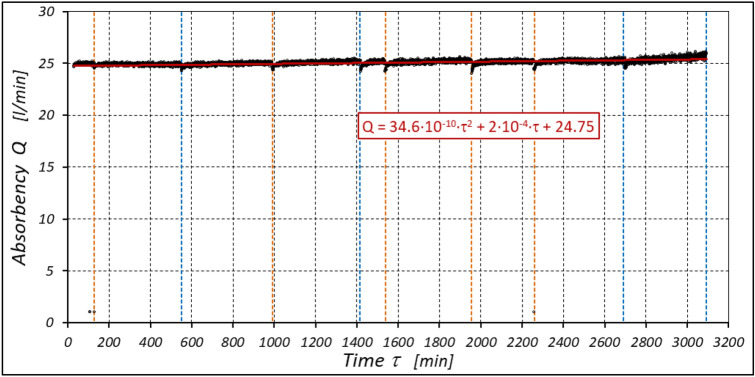
Characteristics of the motor absorbency Q as a function of time τ for Δp = 15 MPa and n = 1500 rpm.

**Figure 22:**
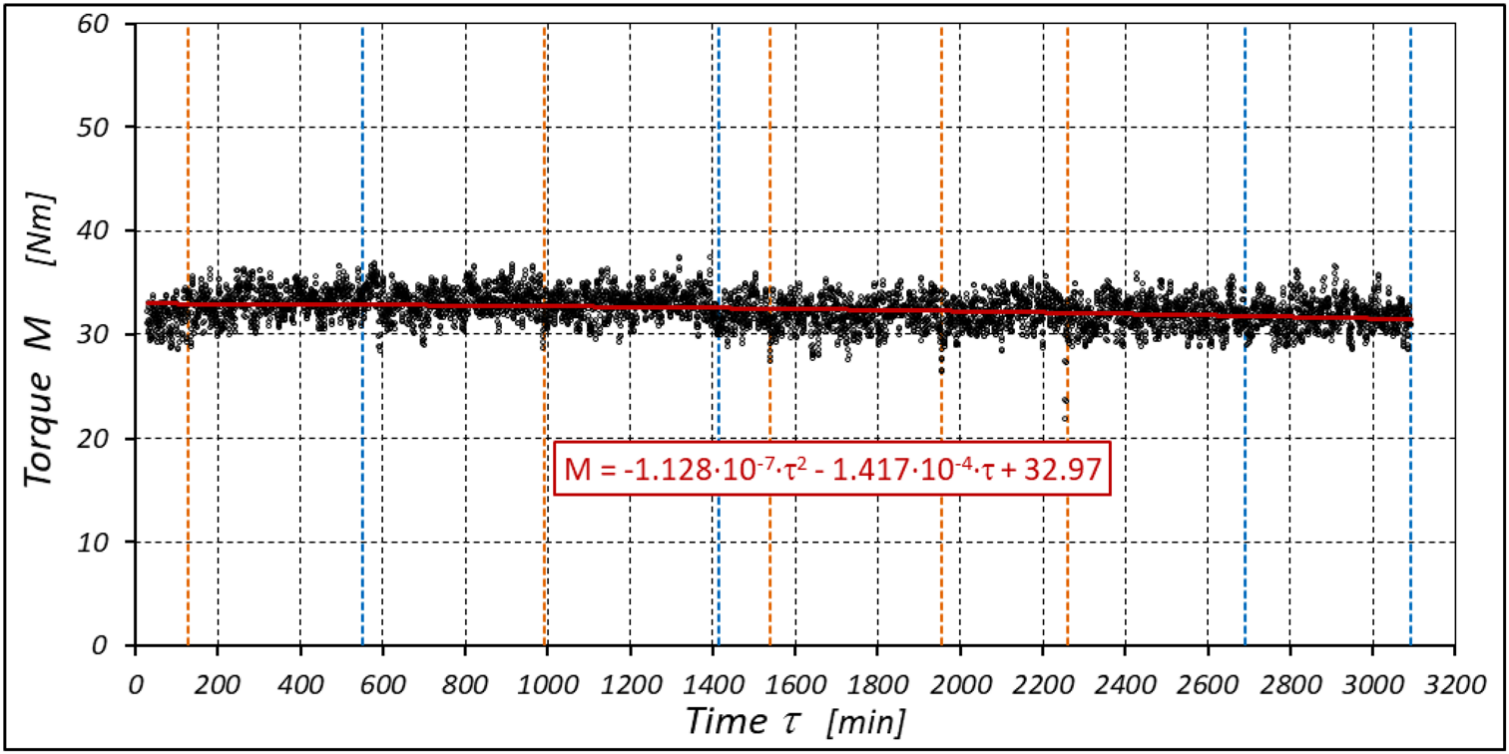
Characteristics of the torque M as a function of time τ for Δp = 15 MPa and n = 1500 rpm.

**Figure 23:**
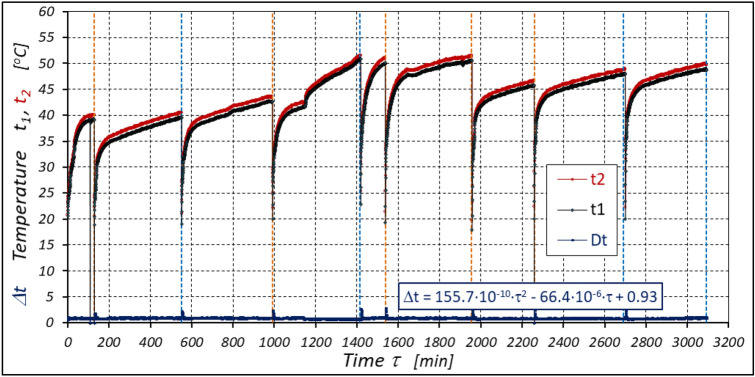
Characteristics of the temperatures t_1_ and t_2_ in the motor ports as a function of time τ for Δp = 15 MPa and n = 1500 rpm.

The results of the measurements of the pressures p_1_ and p_2_, the absorption Q, the torque M and the temperatures t_1_ and t_2_ at the motor ports are shown in Figs. 20, 21, 22 and 23.

After 3092.55 min of operation, the satellite mechanism was destroyed. The process of wear of the mechanism components until failure was similar to the tests for Δp = 20 MPa (Figs. 17, 18 and 19).

### *Results of the test at Δp* = *10 MPa*

Table 5 shows the mass of the components of the satellite mechanism, the height H_E_ of the curvature and the average values h_R_ of the axial clearances of the rotor and the average values h_S_ of the axial clearances of the satellite measured before, during and after the tests.

**Table 5:** Mass, height and clearances of the componensts of the satellite mechanism—test for Δp = 10 MPa. Symbols H_E_, h_R_ and h_S_ like in Fig. 10.

		m_E_ (g)	m_R_ (g)	m_S_ (g)	H_E_ (mm)	h_R_ (µm)	h_S_ (µm)
Time τ(min)	0	99.650	43.054	4.532	19.895	5.5	5.8
	1408	99.610	43.005	4.522	19.902	6.1	6.1
	1814	99.591	42.940	4.520	19.923	30.5	32.15
	3237	99.532	42.896	4.511	19.922	29.0	32.4
	4797	99.423	42.809	4.495	19.934	39.5	41.6
	6652*	99.315	42.715	4.473	19.930	39.0	40.5

*The mechanism is not broken!

**Figure 24:**
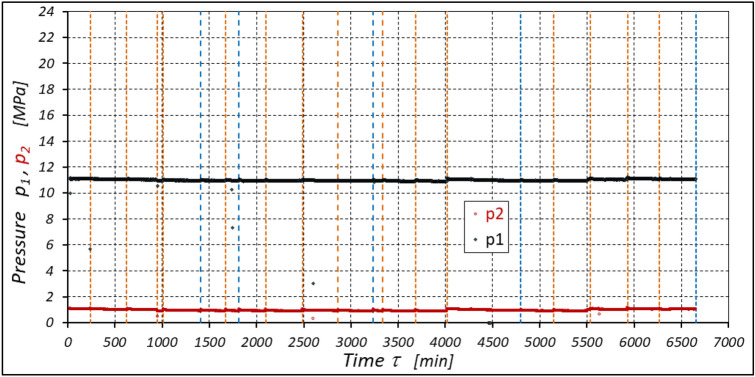
Characteristics of the pressure p_1_ and p_2_ in the motor ports as a function of time τ for Δp = 10 MPa and n = 1500 rpm.

**Figure 25:**
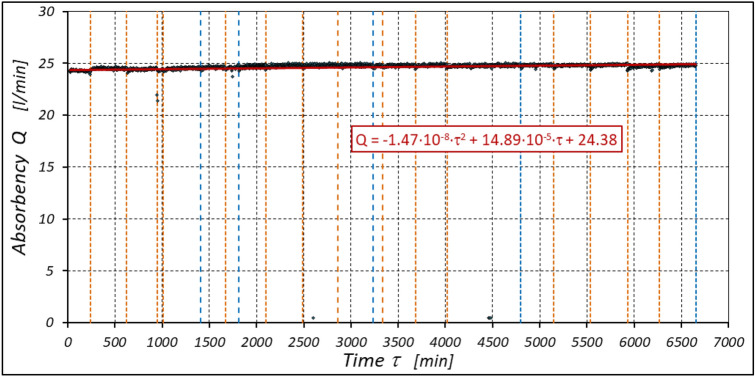
Characteristics of the motor absorbency Q as a function of time τ for Δp = 10 MPa and n = 1500 rpm.

**Figure 26:**
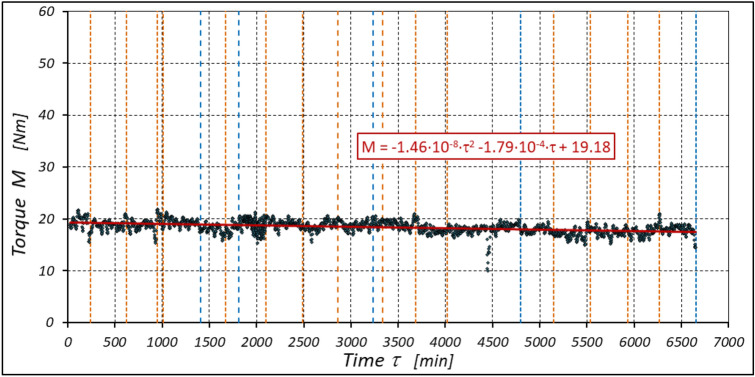
Characteristics of the torque M as a function of time τ for Δp = 10 MPa and n = 1500 rpm.

**Figure 27:**
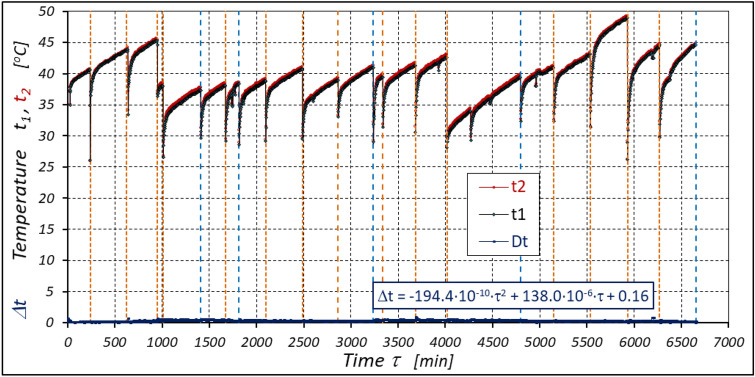
Characteristics of the temperatures t_1_ and t_2_ in the motor ports as a function of time τ for Δp = 10 MPa and n = 1500 rpm.

The results of the measurements of the pressures p_1_ and p_2_, the absorption Q, the torque M and the temperatures t_1_ and t_2_ at the motor ports are shown in Figs. 24, 25, 26 and 27.

**Figure 28:**
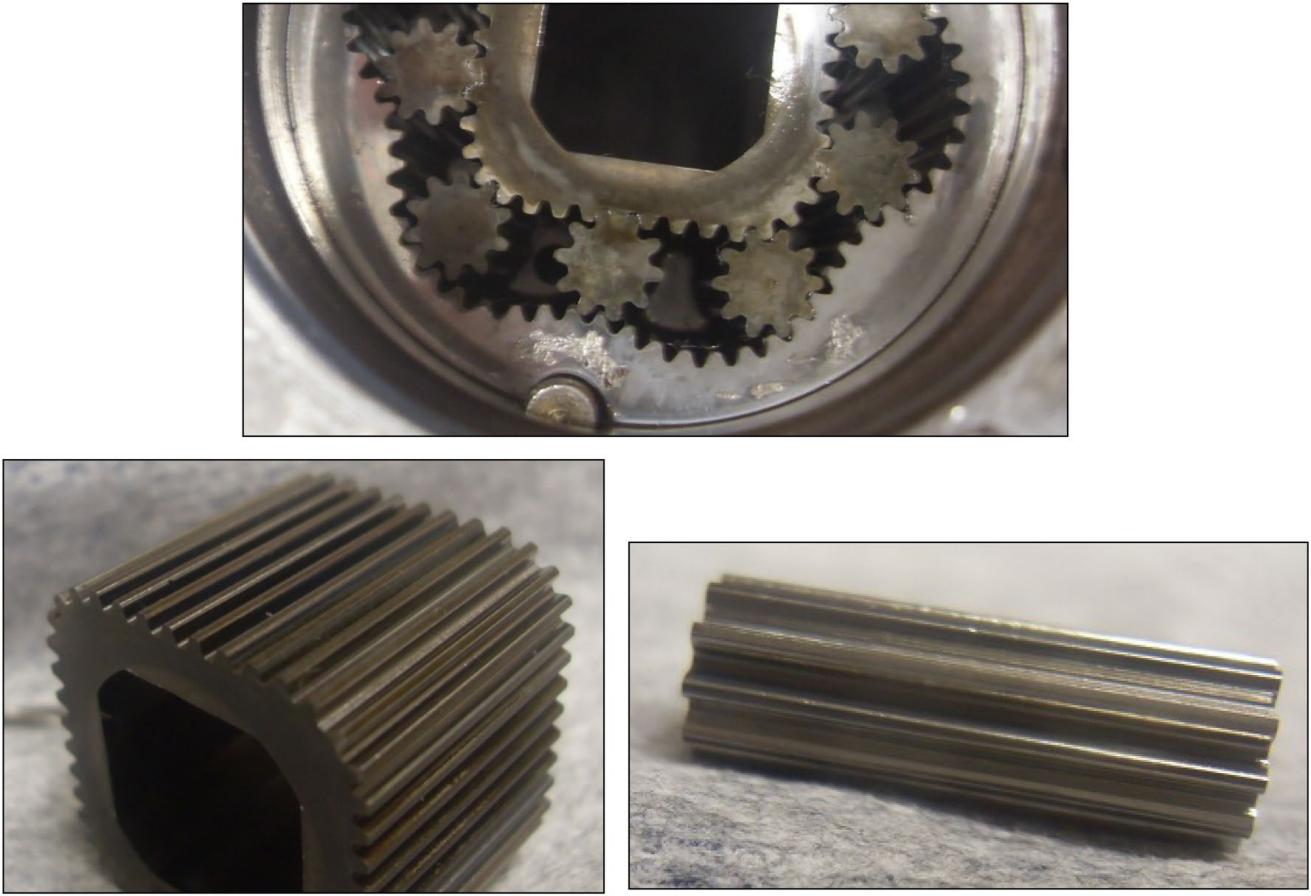
Satellite mechanism after the test at Δp = 10 MPa.

After more than 110 h (6652 min –Table 5) of testing, the motor was disassembled and no damage to the components of the mechanism visible to the naked eye was found (Fig. 28). Only an increase in satellite clearances in the mechanism and pitting on the curvature and on the commutation plates were visible. The test of the motor was terminated at this stage.

**Figure 29:**
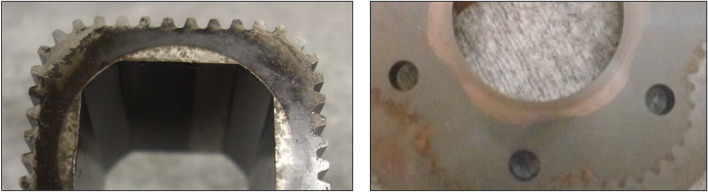
A layer of burnt rapeseed oil on the rotor surface (left) and the characteristic purple layer on the commutation plate (right) —after 3237 min of operation.

The publication^42^ states that during the first tests of the satellite motor supplied with rapeseed oil, a phenomenon was observed, namely a black ring on the face of the rotor and a purple ring on the commutation plate at the point of interaction with the rotor. This was also the case here, i.e. during tests at Δp = 10 MPa. After each disassembly of the satellite mechanism, both the black ring on the rotor surface and the purple ring on the commutation plate were also observed (Fig. 29). It was found that the thickness of the ring on the rotor is close to the height of the gap between the rotor and the commutation plate.

It is significant that these rings were not present when testing the motor with higher pressure drop, i.e. at Δp = 15 MPa and at Δp = 20 MPa.

## Discussion

**Figure 30:**
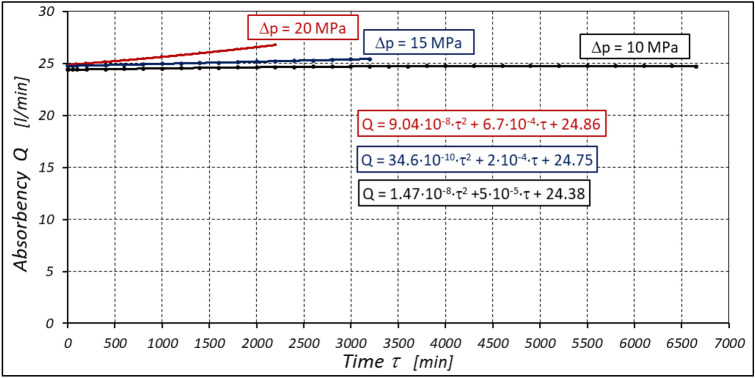
Characteristics of the motor absorbency Q as a function of time τ for different fixed pressure drop Δp in the motor.

**Figure 31:**
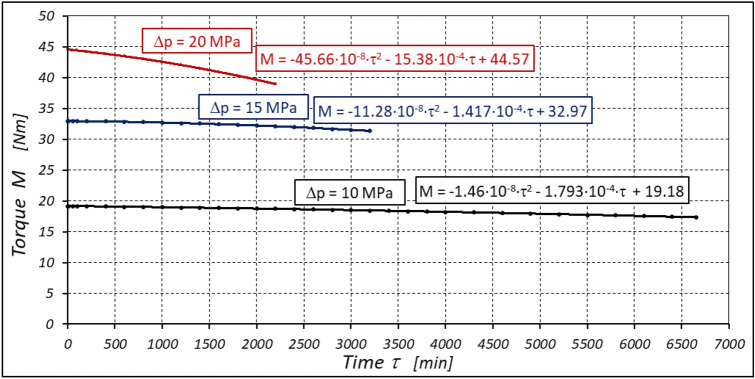
Characteristics of the torque M as a function of time τ for different fixed pressure drop Δp in the motor.

The test results have shown that in the first minutes of the motor operation (the non-worn mechanisms):

- the absorption Q of the motor is largest (24.9 l/min) for the highest pressure drop in the motor (Δp = 20 MPa) and smallest (24.4 l/min) for the lowest pressure drop (Δp = 10 MPa) (Fig. 30). This is consistent with the flow theory for hydraulic motor described in^39^;
- similarly, the torque M on the motor shaft is the highest (44.6 Nm) for the highest pressure drop in the motor (Δp = 20 MPa) and lowest (19.2 Nm) for the lowest pressure drop (Δp = 10 MPa) (Fig. 31). This agrees with the theory that describes the influence of the load (torque) M on the pressure drop Δp in the motor, as described in^40^ and shown in formulae (5) and (6).

**Figure 32:**
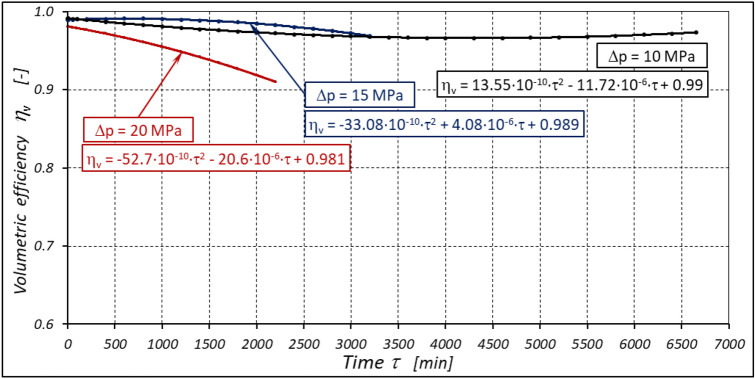
Volumetric efficiency h_v_ of the motor as a function of time τ for different fixed pressure drop Δp in the motor.

With the hydraulic motor operation time τ (at n = const.), the motor absorbency Q increases and the torque M decreases (Figs. 30 and 31). The greatest increase in absorption Q is observed for the most heavily loaded motor (Fig. 30). Therefore, an increase in absorbency means a lower volumetric efficiency η_v_ of the motor (Fig. 32). The increase in the absorbency Q of the motor also means an increase in the pressure drop Δp_ich_ in the internal channels of this motor, which in turn, at Δp = const., causes a smaller pressure drop Δp_i_ in the working chambers, i.e. Δp_i_ < Δp (according to formula (6)). For absorbency Q = 24.4 ÷ 24.9 l/min is Δp_ich_ ≈ 1.4 MPa (determined according to the method described in^49^). So, according to formula (6) the following pressure difference occurs in the chambers of the working mechanism:

- Δp_i_ ≈ 18.6 MPa for Δp = 20 MPa;
- Δp_i_ ≈ 13.6 MPa for Δp = 15 MPa;
- Δp_i_ ≈ 8.6 MPa for Δp = 10 MPa.

**Figure 33:**
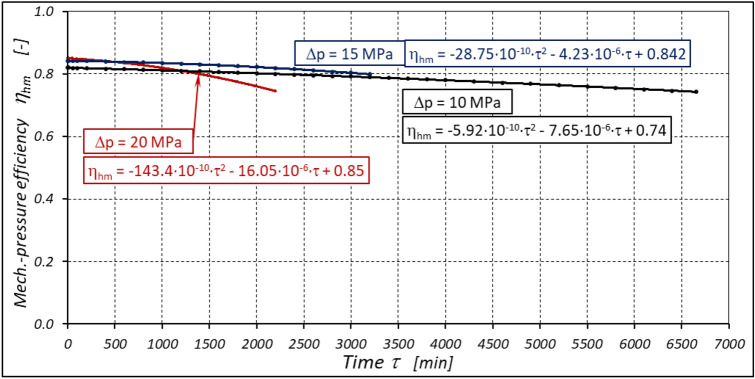
Mechanical-pressure efficiency h_hm_ of the motor as a function of time τ for different fixed pressure drop Δp in the motor.

The result is a lower value of the torque M measured on the motor shaft (Fig. 31) and a lower mechanical-pressure efficiency η_hm_ (Fig. 33).

According to Figs. 32 and 33 the value of the volumetric efficiency η_v_ and the mechanical-pressure efficiency η_hm_ changes during the time τ of motor operation as follows:

a) for Δp = 10 MPa:

22 $${\eta }_{v}=0.99+13.55\cdot {10}^{-10}\cdot {\tau }^{2}-11.72\cdot {10}^{-6}\cdot \tau$$

23 $${\eta }_{hm}=0.74-5.92\cdot {10}^{-10}\cdot {\tau }^{2}-7.65\cdot {10}^{-6}\cdot \tau$$

b) for Δp = 15 MPa:

24 $${\eta }_{v}=0.989-33.08\cdot {10}^{-10}\cdot {\tau }^{2}+4.08\cdot {10}^{-6}\cdot \tau$$

25 $${\eta }_{hm}=0.842-28.75\cdot {10}^{-10}\cdot {\tau }^{2}-4.23\cdot {10}^{-6}\cdot \tau$$

c) for Δp = 20 MPa:

26 $${\eta }_{v}=0.981-52.7\cdot {10}^{-10}\cdot {\tau }^{2}-20.6\cdot {10}^{-6}\cdot \tau$$

27 $${\eta }_{hm}=0.85-143.4\cdot {10}^{-10}\cdot {\tau }^{2}-16.05\cdot {10}^{-6}\cdot \tau$$

In publications^22,37^ it was shown that for the satellite motor supplied with mineral oil, in the first period of its operation (i.e. for the so-called new motor) there is:

- for Δp = 10 MPa: η_v_ = 0.96 and η_hm_ = 0.73;
- for Δp = 15 MPa: η_v_ = 0.95 and η_hm_ = 0.82;
- for Δp = 20 MPa: η_v_ = 0.94 and η_hm_ = 0.87.

From the test results and the analyses described above, it can be concluded that the partial efficiencies of the satellite motor supplied with rapeseed oil are comparable to those of the motor supplied with mineral oil.

The increase in the absorbency Q of the motor and thus the decrease in the volumetric efficiency η_v_ over time is the result of wear processes in the working mechanism. This is shown in particular by the loss of mass of the mechanism components and the increase in the axial clearance of the rotor and satellites (Tables 3, 4 and Table 5). The characteristics of the absorbency Q and volumetric efficiency hv of the motor at Δp = 10 MPa are quite unexpected (Figs. 30 and 32). The absorbency Q of the motor does not change much as a function of time τ (initially it increases slightly up to t ≈ 3500 min and then decreases slightly—Fig. 30), although the axial clearances of the rotor and the satellites have increased considerably (Table 5).

It was also observed that rapeseed oil sticks to the front surface, especially the rotor (Figs. 28 and 29). Tribological examinations of the materials of the friction pairings lubricated with rapeseed oil showed that it is a film of burnt oil. This burnt oil layer is characterised by very good tribological properties^22,45^. The thickness of this layer was measured and found to be comparable to the height of the gap between the rotor and the commutation plate. Table 5 shows quite large values for the axial clearance of the rotor and the satellites, as these are the values after running-in of the elements (as already written in Sect. 7.3) and therefore do not include a burnt oil layer.

It is significant that at higher pressure drops in the motor, i.e. at Δp > 10 MPa. This can be explained as follows. The flow in the flat gap formed by the rotor and the commutation plate is a function of the pressure drop in this gap^39^. The flow in this gap is the flow from the HPC high pressure chamber to the shaft chamber. The shaft chamber is connected to the HPC low-pressure chamber^22^. Therefore, the pressure drop in this gap is comparable to the pressure drop Δp_i_ in the working chambers.

Therefore, at low Δp_i_ there is a very low value of flow in the gap. In addition, there are errors in the manufacturing of the components of the working mechanism. This means that the rotor surface is not parallel to the commutation plate and there is friction between these elements. As a result, the surface temperature of these elements increases. With a very low liquid flow in the gap, these surfaces do not have time to cool down. Therefore, the oil particles are burnt and a layer of burnt oil forms. With larger pressure drops Δp_i_ in the working chambers, there is a larger flow of liquid in the gap. This is sufficient to prevent an excessive rise in temperature.

The test results confirmed the theoretical analyses described in Sect. 5 regarding the temperature increase Δt in the motor. This Δt (measured in the motor ports) is the result of the energy losses occurring in the motor. Formula (14) shows that the liquid parameters that have a direct impact on the temperature increase in the motor are density ρ and specific heat c. However, the viscosity υ of the liquid has an indirect effect., i.e. it significantly affects the volumetric efficiency η_v_ of the motor, the pressure efficiency η_h_ of the motor and, to a much lesser extent, the mechanical efficiency η_m_ of the motor, which has been demonstrated in the author’s previous publications, including: in^22,39,40^.

The temperature difference Δt in the motor can therefore be an indicator of the total efficiency η_c_. This efficiency can therefore be calculated by transforming formula (14), i.e.:

28 $${\eta }_{c}=1-\rho \cdot c\cdot \left(k+\frac{\Delta T}{\Delta p}\right)$$

**Figure 34:**
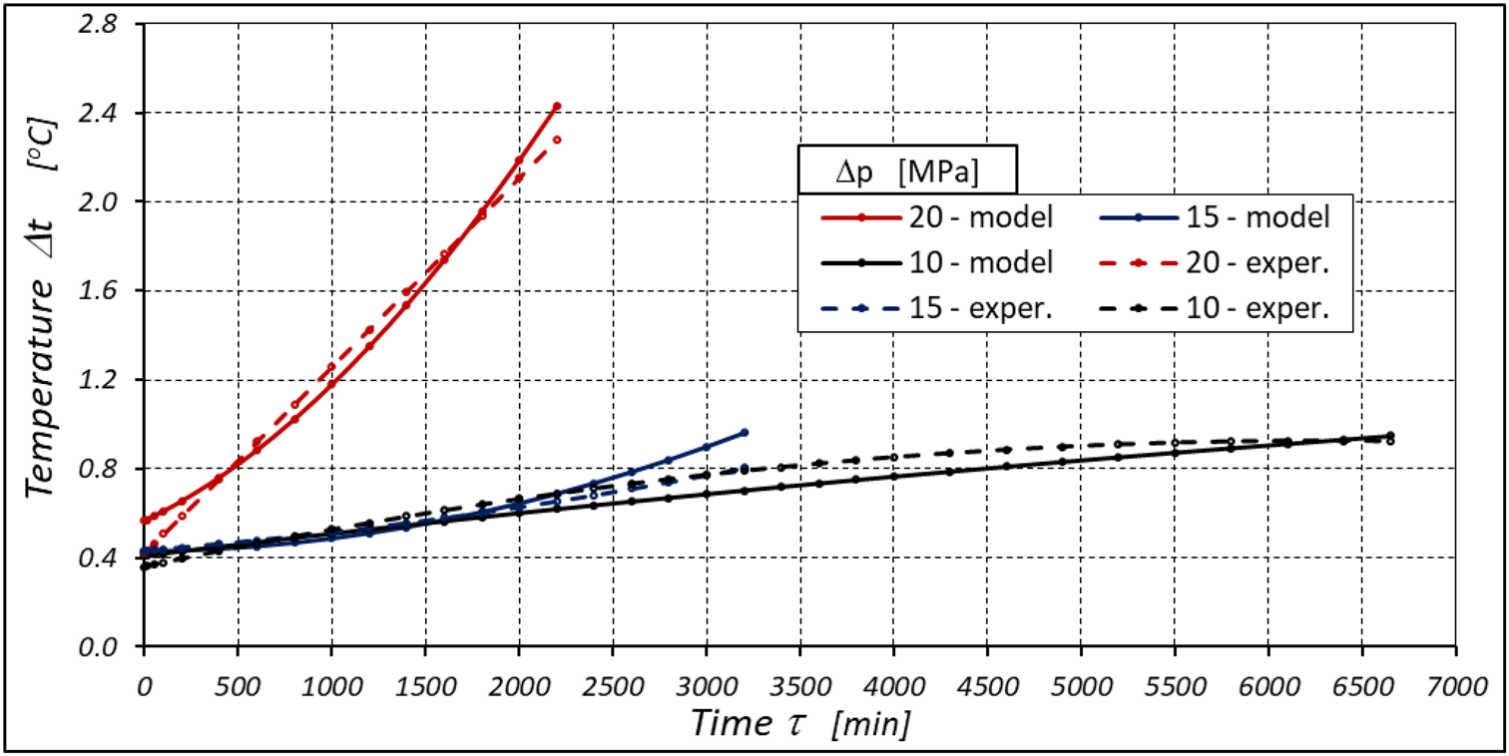
Rise in temperature Δt in the motor as a function of the motor operation time τ calculated according to formula (14) and the result of the experiment.

As there is no information in the literature regarding the value of the k constant for mineral oil, the value of this constant was calculated according to formula (16), where the efficiency values hv and hhm and rise in temperature Δt were calculated according to formulas (22) ÷ (31). In this way, k = 0.071 °C /MPa was determined. This value appears to be quite realistic. For comparison, the value k given in^52^ for Hydrol mineral oil at the same temperature is k = 0.128 °C /MPa. For comparison purposes, Fig. 34 shows the rise in temperature Δt in the motor, which was calculated according to formula (14) (for k = 0.071 °C /MPa) and obtained from the experiment.

The differences between the calculated and measured temperatures are not large. Therefore, these differences do not disqualify the tests described above. Therefore, the lower the total efficiency η_c_ of the motor, the greater the rise in temperature Δt in the motor. Measuring the temperature difference Δt in the motor can therefore be an indicator of the overall efficiency of the motor.

The experimental data (Figs. 14, 22 and 26) show that the rise in temperature Δt changes during the time τ of motor operation as follows:

a) for Δp = 10 MPa:

29 $$\Delta t=0.16-194.4\cdot {10}^{-10}\cdot {\tau }^{2}+138.0\cdot {10}^{-6}\cdot \tau$$

b) for Δp = 15 MPa:

30 $$\Delta t=0.93+155.7\cdot {10}^{-10}\cdot {\tau }^{2}-66.4\cdot {10}^{-6}\cdot \tau$$

c) for Δp = 20 MPa:

31 $$\Delta t=0.42+61.5\cdot {10}^{-10}\cdot {\tau }^{2}+828.6\cdot {10}^{-6}\cdot \tau$$

**Figure 35:**
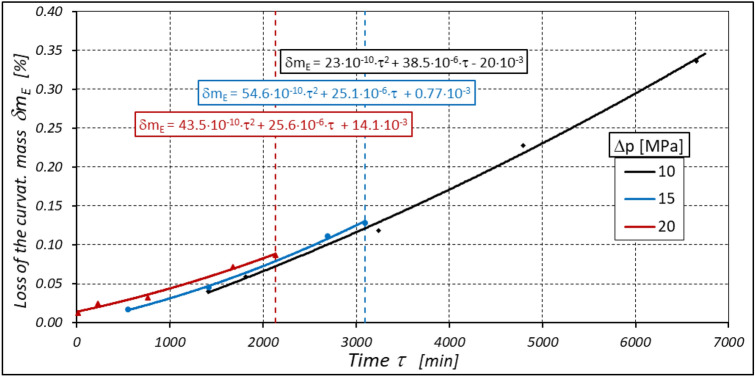
Percentage loss δm_E_ of the curvature mass as a function of time τ.

**Figure 36:**
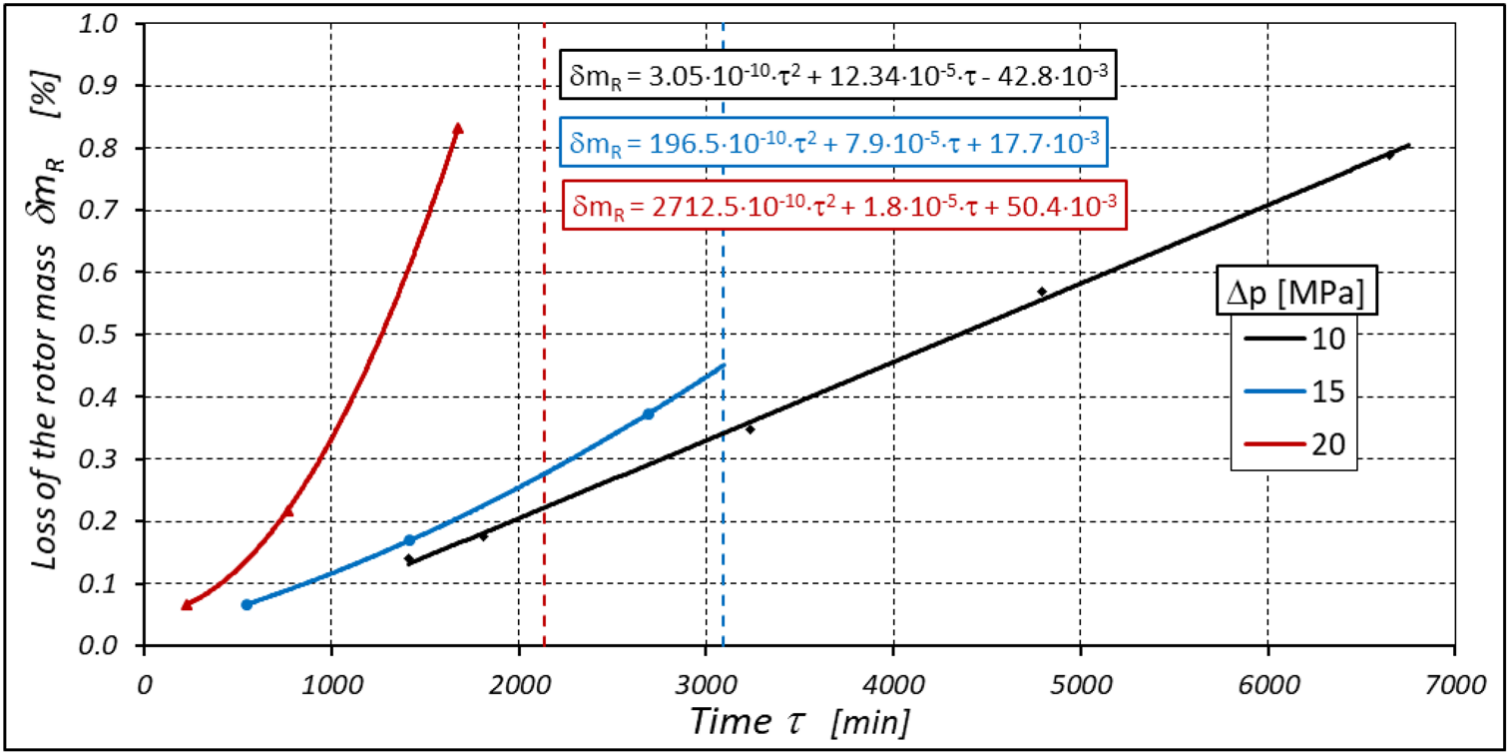
Percentage loss δm_R_ of the rotor mass as a function of time τ.

**Figure 37:**
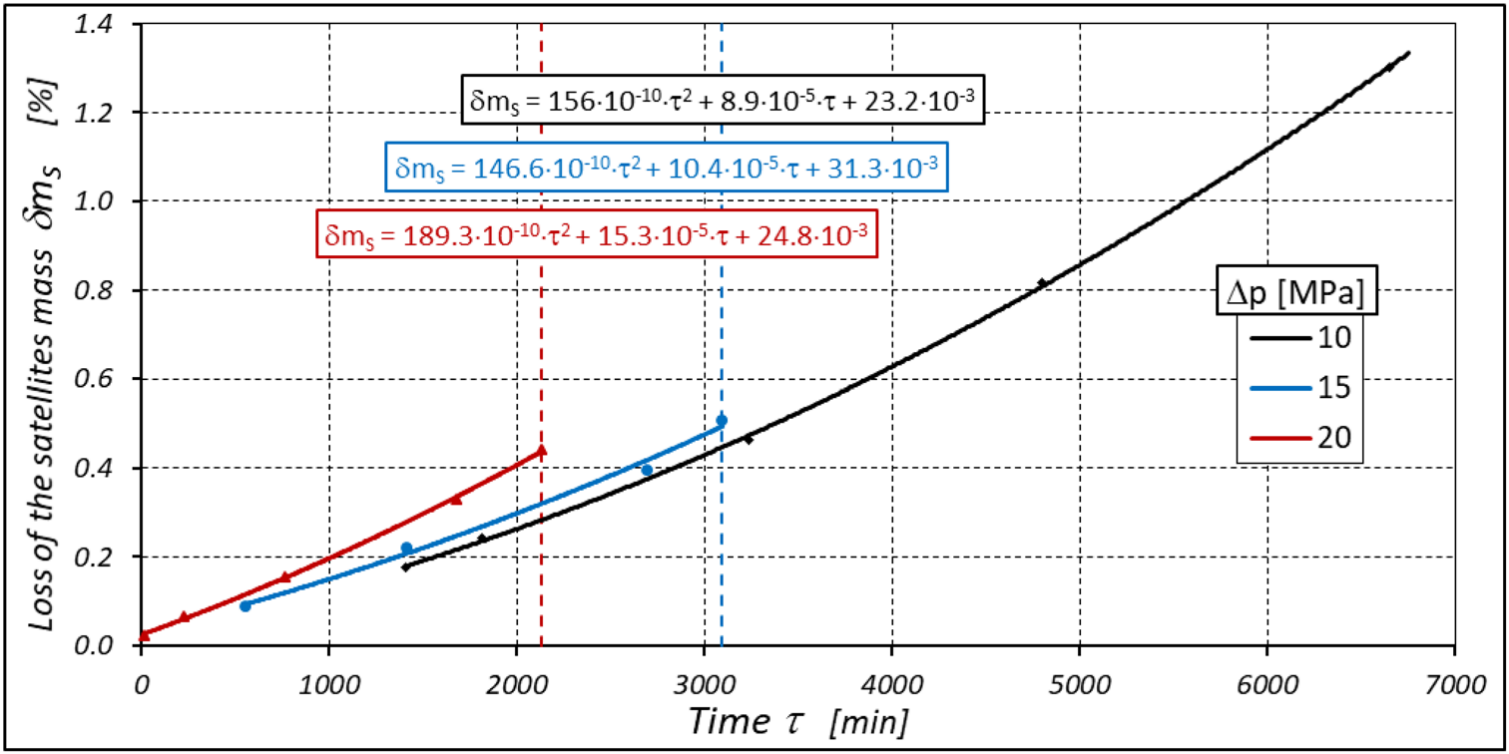
Percentage loss δm_S_ of the satellites mass as a function of time τ.

As already mentioned abrasive wear of the gear elements of the working mechanism also occurs during motor operation, resulting in weight loss of these elements. The greatest and fastest weight loss of these elements occurs at the highest motor loads (Figs. 35, 36 and 37).

**Figure 38:**
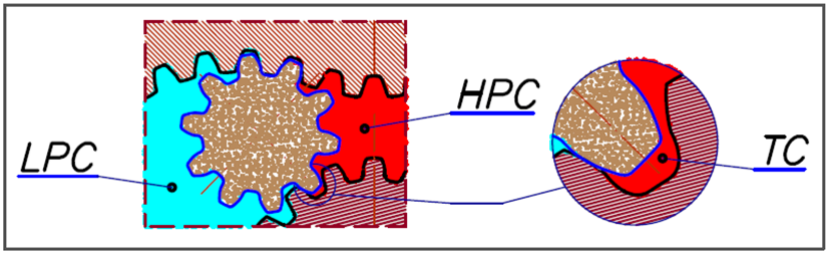
Unfavourable position of the satellite in relation to the rotor—large intertoothed and tip clearances of the interacting teeth.

Furthermore, the characteristics presented in Figs. 35, 36 and 37 show that the rotor has the greatest relative weight loss, especially at Δp ≥ 15 MPa. The reasons for this are as follows:

1. during one complete revolution of the rotor, each rotor tooth is loaded the greatest number of times (see Sect. 2 and^38^);
2. the change in the direction of tooth loadin that occur during the phase change of the chambers adjacent to the satellite (i.e. during the transition of the chamber from the high-pressure chamber to the low-pressure chamber and vice versa);
3. the presence of a centrifugal force acting on the satellite. Its effect is the disappearance of the clearances in the co-operation between the satellite and the bypass and the increase in the clearances in the co-operation between the satellite and the rotor, which is shown in Figs. 28 and 38. Therefore, in the area of co-operation between the satellite teeth and the rotor teeth, the conditions for co-operation deteriorate;
4. within the inter-tooth clearance, there is a phenomenon of the satellite tooth hitting the rotor tooth when the pressure in the chamber changes from low to high^38^.

Due to the phenomena described above, the rotor wears out the fastest. The curvature wears the slowest because it has the most favourable working conditions.

During the motor operation time τ, until the mechanism is destroyed, a certain number of load cycles occur on each curvature tooth, rotor and satellite. This number can be calculated using the following formula:

a) for the satellite tooth:

32 $${{\text{i}}}_{{\text{dS}}}={{\text{i}}}_{{\text{S}}}\cdot \left({\text{n}}\cdot\uptau +{{\text{n}}}_{{\text{m}}}\cdot {\uptau }_{{\text{s}}}\right)$$

b) for the rotor tooth:

33 $${{\text{i}}}_{{\text{dRTS}}}={{\text{i}}}_{{\text{RTS}}}\cdot \left({\text{n}}\cdot\uptau +{{\text{n}}}_{{\text{m}}}\cdot {\uptau }_{{\text{s}}}\right)$$

c) for the curvature tooth:

34 $${{\text{i}}}_{{\text{dETS}}}={{\text{i}}}_{{\text{ETS}}}\cdot \left({\text{n}}\cdot\uptau +{{\text{n}}}_{{\text{m}}}\cdot {\tau }_{{\text{s}}}\right)$$

where the set-up time τ_s_ and the mean rotational speed n_m_ were described in Sect. 6.

An overview of the number of cycles i_d_ for each element of the mechanism from all test series is shown in Table 6.

**Table 6:** The number of load cycles of the teeth until their destruction for n = 1500 rpm and n_m_ = 750 rpm.

	τ (min)	τ_s_ (min)	i_S_	i_RTS_	i_ETS_	i_dS_	i_dRTS_	i_dETS_
Δp (MPa)								
10*	6652*	52	5.28	6	4	52,889,760	60,102,000	40,068,000
15	3092.5	30				24,611,400	27,967,500	18,645,000
20	2135	40				17,067,600	19,395,000	12,930,000

*The mechanism is not destroyed!

From the results in the above table, it can be concluded that:

- the rotor teeth are characterised by the highest number of load cycles;
- the durability of the working mechanism for Δp > 10 MPa is very low (approx. 51.5 h for Δp = 15 MPa and 35.5 h for Δp = 20 MPa);
- the durability of the working mechanism cannot be accurately determined for Δp = 10 MPa, as the test was cancelled after more than 110 h of motor operation;
- to achive satisfactory durability of the satellite mechanism, the motor should be operated at Δp < 10 MPa;
- the results of the theoretical analyzes contained in^38^ were confirmed, i.e. the working mechanism is overloaded (especially at Δp = 20 MPa) and the stresses in the teeth of this mechanism exceed the permissible values.

A significant observed phenomenon in each series of tests is the increase in the height of the curvature and the increase in the axial clearance of the rotor and satellites (Tables 3, 4 and 5). It can be seen that this phenomenon correlates with the phenomenon of fretting on the end surfaces of the curvature and the commutation plates (Figs. 17, 18 and 28). The reason for this phenomenon is the low stiffness of the curvature. When the pressure drop Δp_ich_ in the working chambers increases, the actual working volume of the mechanism increases, which has been demonstrated in publications^37,47,48^. Therefore, elastic deformation of both the curvature and the commutation plates must occur, which is evidenced by the visual effect, i.e. fretting. As a result of fretting, detached particles of the curvature material and the commutation plates can be hammered into the surface, especially of the curvature in the immediate vicinity of the fretting centre.

The curvature is an element made of a material that has a lower hardness than the commutation plate (sintered carbide). This create bumps on the curvature that increase its height. During periodic disassembly of the motor, these bumps were carefully removed by lapping. However, care was taken not to break in too intensively so as not to reduce the height of the curvature and thus not to eliminate the axial clearances of the satellites and the rotor. Therefore, these bumps were not completely removed, which led to an increase in the axial clearance.

## Final conclusion

The test results of a satellite motor supplied with rapeseed oil have proven that:

1. the satellite motor can be supplied with rapeseed oil;
2. the efficiencies of the tested motor are comparable to the efficiencies of the motor supplied with mineral oil;
3. the durability of the satellite mechanism strongly depends on the pressure drop Δp_i_ in the working chambers, and thus depends on the motor load M and its absorbency Q;
4. the satellite mechanism is generally characterised by very low durability at a pressure drop higher than 10 MPa. The research results confirmed the general conclusion from the theoretical analyses described in^38^, i.e. ‘satellite positive displacement machines (pumps, motors) should operate at low speed and at most an average working pressure”;

From the research results presented in this article, it can also be concluded that it is necessary to develop a tooth profile (shape) other than that of the involute and characterised by lower stresses in the area of cooperation. This procedure would have a positive effect on extending the operating time of the satellite mechanism and thus on its durability, especially under high loads M.

## Data Availability

The datasets used and/or analysed during the current study are available from the corresponding author on reasonable request.
